# Single Atom Engineered Antibiotics Overcome Bacterial Resistance

**DOI:** 10.1002/adma.202410652

**Published:** 2024-09-23

**Authors:** David Panáček, Jan Belza, Lucie Hochvaldová, Zdeněk Baďura, Giorgio Zoppellaro, Martin Šrejber, Tomáš Malina, Veronika Šedajová, Markéta Paloncýová, Rostislav Langer, Lukáš Zdražil, Jianrong Zeng, Lina Li, En Zhao, Zupeng Chen, Zhiqiang Xiong, Ruibin Li, Aleš Panáček, Renata Večeřová, Pavla Kučová, Milan Kolář, Michal Otyepka, Aristides Bakandritsos, Radek Zbořil

**Affiliations:** ^1^ Regional Centre of Advanced Technologies and Materials Czech Advanced Technology and Research Institute (CATRIN) Palacký University Olomouc Šlechtitelů 241/27 Olomouc‐Holice 783 71 Czech Republic; ^2^ Nanotechnology Centre Centre for Energy and Environmental Technologies VŠB–Technical University of Ostrava 17. listopadu 2172/15 Ostrava‐Poruba 708 00 Czech Republic; ^3^ Department of Physical Chemistry Faculty of Science Palacký University Olomouc 17. listopadu 1192/12 Olomouc 771 46 Czech Republic; ^4^ IT4Innovations VŠB‐Technical University of Ostrava 17. listopadu 2172/15 Ostrava‐Poruba 708 00 Czech Republic; ^5^ Shanghai Synchrotron Radiation Facility Shanghai Advanced Research Institute Chinese Academy of Sciences Shanghai 201204 P. R. China; ^6^ Jiangsu Co‐Innovation Center of Efficient Processing and Utilization of Forest Resources International Innovation Center for Forest Chemicals and Materials College of Chemical Engineering Nanjing Forestry University Longpan Road 159 Nanjing 210037 P. R. China; ^7^ State Key Laboratory of Radiation Medicine and Protection School for Radiological and Interdisciplinary Sciences (RAD‐X) Collaborative Innovation Center of Radiation Medicine of Jiangsu Higher Education Institutions Suzhou Medical College Soochow University Suzhou Jiangsu 215123 P. R. China; ^8^ Department of Microbiology Faculty of Medicine and Dentistry Palacký University Olomouc Hněvotínská 3 Olomouc 779 00 Czech Republic

**Keywords:** antibiotic, cytocompatibility, manganese, multi‐drug resistance, single‐atom

## Abstract

The outbreak of antibiotic‐resistant bacteria, or “superbugs”, poses a global public health hazard due to their resilience against the most effective last‐line antibiotics. Identifying potent antibacterial agents capable of evading bacterial resistance mechanisms represents the ultimate defense strategy. This study shows that –the otherwise essential micronutrient– manganese turns into a broad‐spectrum potent antibiotic when coordinated with a carboxylated nitrogen‐doped graphene. This antibiotic material (termed NGA‐Mn) not only inhibits the growth of a wide spectrum of multidrug‐resistant bacteria but also heals wounds infected by bacteria in vivo and, most importantly, effectively evades bacterial resistance development. NGA‐Mn exhibits up to 25‐fold higher cytocompatibility to human cells than its minimum bacterial inhibitory concentration, demonstrating its potential as a next‐generation antibacterial agent. Experimental findings suggest that NGA‐Mn acts on the outer side of the bacterial cell membrane via a multimolecular collective binding, blocking vital functions in both Gram‐positive and Gram‐negative bacteria. The results underscore the potential of single‐atom engineering toward potent antibiotics, offering simultaneously a long‐sought solution for evading drug resistance development while being cytocompatible to human cells.

## Graphical abstract

Single atoms of manganese, tightly bonded to nitrogen‐doped carboxylated graphene, exhibit potent antibacterial properties and bypass bacterial resistance development, even after thirty bacterial generations. Antibacterial activity is observed against both Gram‐negative and Gram‐positive bacteria, as well as in in‐vivo models, while the material is nontoxic to human cells.

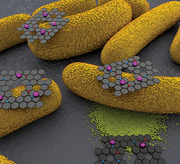

## 1 Introduction

The ability of bacteria to develop multi‐drug resistance (MDR) to antibiotics threatens one of the pillars of modern medicine and breakthrough discoveries of the last century against bacterial infections.^[^ ^1^, ^2^ ^]^ A global report by the World Health Organization (WHO) estimates that unless new antibacterial drugs are discovered, no effective antibiotics will be available by 2050, and if MDR continues to grow at the same rate, bacterial infections will become the leading cause of death by 2050, with an estimated economic cost of 100 trillion USD.^[^ ^3^, ^4^ ^]^ It is, therefore, essential to address this issue systematically; otherwise, the likelihood of returning to the pre‐antibiotic era may become alarmingly high.^[^ ^5^ ^]^ Hence, research endeavors are directed toward identifying the next generation of highly active antibacterial (bio)molecules^[^ ^6^, ^7^, ^8^, ^9^, ^10^, ^11^ ^]^ and nanomaterials.^[^ ^12^, ^13^, ^14^, ^15^ ^]^

Current antibacterial research on natural or modified proteins,^[^ ^16^ ^]^ peptides,^[^ ^7^, ^8^, ^9^, ^10^, ^11^ ^]^ synthetic small‐molecules,^[^ ^6^, ^17^ ^]^ and natural toxins^[^ ^18^ ^]^ has generated significant momentum in the field. For example, modified arylomycin peptides exhibit potent activity against strains where natural analogs are ineffective.^[^ ^7^ ^]^ They are benign to normal cells, and display low frequency of MDR development, as identified after treatment of one bacterial generation for 48 h. Additional serial passages will be required to address the persistence of their activity because of the known resistance development to arylomycin's targets via mutations^[^ ^19^ ^]^ arising from bacterial mechanisms that are well‐adapted against both molecular threats and toxic ions.^[^ ^20^, ^21^ ^]^ A proline‐rich protein was also recently discovered^[^ ^16^ ^]^ with high efficacy against bacteria. Although promising and, possibly, non‐toxic upon administration of antibacterial doses, the protein was active only against single‐membrane Gram‐positive bacteria. Its response regarding resistance development during sequential treatment of many bacterial generations remains unknown. Potent antimicrobial stapled peptides,^[^ ^11^ ^]^ modified natural lipopeptides,^[^ ^8^ ^]^ and synthetic molecules^[^ ^17^ ^]^ were also developed, but their effectiveness in escaping MDR must be investigated as well. A naturally inspired lipopeptide, cilagicin, with the ability to escape MDR after 25 serial bacterial passages,^[^ ^10^ ^]^ showed high activity only against Gram‐positive strains. These endeavors demonstrate the progress, significance, and obstacles in uncovering efficacious antibiotics, particularly in the context of the remarkable ability of bacteria to bypass their action.^[^ ^20^, ^22^ ^]^

To this end, the development of (nano)materials^[^ ^15^ ^]^ such as inorganic^[^ ^23^, ^24^ ^]^ and carbon‑based nanomaterials,^[^ ^25^, ^26^, ^27^, ^28^ ^]^ metals,^[^ ^29^ ^]^ polymers,^[^ ^30^, ^31^ ^]^ charge‐^[^ ^28^ ^]^ and light‑activated nano‐systems^[^ ^12^, ^23^, ^32^ ^]^ emerge as an alternative tool for the treatment of infectious diseases. Pristine graphene and related materials have been explored in this direction, with their activity being dependent on the application pathway (e.g., antibacterial activity studies in Petri dish or in suspension), and the presence of impurities from different synthetic pathways^[^ ^33^, ^34^ ^]^ Often, several dozens of mg of material per liter are required for, mainly, partial bacterial growth inhibition.^[^ ^35^ ^]^ Investigations have also suggested a small^[^ ^35^ ^]^ antibacterial activity of GO if it is well‐purified from metal ions.^[^ ^36^ ^]^ Results from our team on antibacterial testing in suspension of 3 different commercially supplied GO materials and another nitrile‐functionalized graphene derivative^[^ ^37^ ^]^ also did not demonstrate antibacterial activity up to 1500 mg L^−1^.

Silver nanoparticles (Ag NPs) remain one of the most potent antibacterial nano‐agents known to date, as they are highly effective against a wide range of multidrug‐resistant bacteria, exhibiting low minimum concentrations for full‐growth inhibition (MIC_100_ of 1‐15 mg L^−1^).^[^ ^24^, ^38^ ^]^ Nevertheless, Ag NPs have high non‐specific toxicity and we recently reported that bacteria can develop resistance even to Ag NPs by producing flagellin, a protein that triggers their aggregation and deactivation.^[^ ^38^ ^]^ Such bacterial defense mechanisms could be circumvented either chemically, by adding flagellin‐production molecular inhibitors,^[^ ^38^ ^]^ or by engineering strong covalent bonds between Ag NPs and the surface of functionalized graphene, thus preventing their aggregation.^[^ ^37^ ^]^ Despite these breakthrough findings, it is imperative to continue research efforts in order to stay ahead of the emergence of bacterial resistance to flagellin inhibitors, while the use of Ag NPs will remain risky for in vivo application due to their toxicity. The research field of advanced nanomaterials is constantly evolving to address the challenges with varying levels of success. For instance, a modified MXene exhibiting a MIC value of 200 mg L^−1^ against *Escherichia coli* and *Staphylococcus aureus*, required light irradiation (generating bactericidal reactive oxygen species), while its cytocompatibility (in terms of applied concentration) was very similar to its MIC.^[^ ^23^ ^]^ Nanocubosomes enclosing lipophilic antibacterial peptides (LL‐37) with a MIC value of 64 mg L^−1^ against *Escherichia coli*, also exhibited the same cytocompatibility as the MIC value, and narrow activity spectrum.^[^ ^39^ ^]^ A reduced MIC value of 25 mg L^−1^ was achieved with core‐shell Pd‐Ir nanostructures, however for 60% inhibition of bacterial growth, and unknown cytocompatibility.^[^ ^40^ ^]^

The advancement of single‐atom engineering (SAE) holds great promise for the development of materials with previously unattainable properties and broad application potential in catalysis,^[^ ^41^ ^]^ energy storage,^[^ ^42^, ^43^ ^]^ and biomedicine.^[^ ^44^, ^45^ ^]^ One of the most essential aspects of SAE is the discovery of active supports with abundant and controlled metal coordination sites for the anchoring of single atoms (SA). Along with other properties, such as conductivity and biocompatibility, the supports define and control the final nature and function of the SAs, including their firm binding, oxidation states, spatial density, and multi‐level cooperativity.^[^ ^46^ ^]^ Supports engineered from graphene‐based nanomaterials are particularly attractive because they are lightweight, composed of abundant and non‐toxic elements, offer high surface area, conducting or semiconducting properties,^[^ ^47^ ^]^ and have good stability. Furthermore, graphene enables efficient charge transport between the metal atoms and the support, stabilizing rare oxidation states.^[^ ^48^ ^]^ So far, the available coordination sites for SAs in carbon‐based supports have been mainly limited to heteroatom‐doped carbon vacancies,^[^ ^49^, ^50^ ^]^ restricting the tunability of the coordination environment. Another limitation of such porphyrinic‐based motifs is the saturated ligand sphere of SAs, which can be further blocked by the high tendency of graphenic units to stack together. As a result, SAE has so far offered antibacterial materials functioning only via intermediate pathways, such as the production of highly reactive and toxic species (e.g., hydroxyl radicals or superoxide^[^ ^45^, ^51^, ^52^, ^53^, ^54^ ^]^) and application of laser light for photothermally enhanced antibacterial activity.^[^ ^55^, ^56^ ^]^ At the same time, SAE against multidrug‐resistant strains, such as methicillin‐resistant *Staphylococcus aureus*, has been only effective by simultaneous use of hydrogen peroxide.^[^ ^51^ ^]^ Therefore, our society urgently requires antibiotics that are potent (low MIC values against bacteria and MDR strains), safe against normal cells, and impervious to bacterial resistance development.

**Figure 1:**
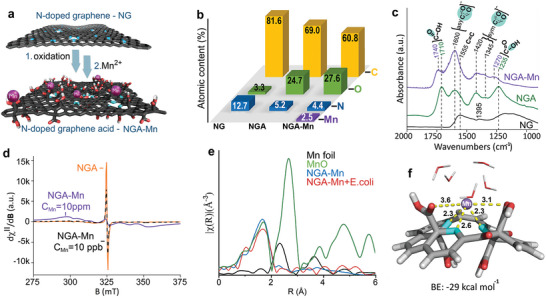
a) Scheme of the NGA‐Mn synthesis (carbon – dark gray; oxygen – red; nitrogen – blue; manganese – magenta). b) Atomic contents of the nitrogen doped‐graphene (NG) after its treatment with nitric acid (NGA) and after immobilization of Mn^2+^ ions (NGA‐Mn). c) FT‐IR spectra of NG, NGA, and NGA‐Mn. d) EPR spectra of NGA, NGA with 10 ppm of Mn^2+,^ and NGA with 10 ppb of Mn^2+^. e) *k* ^2^‐weighted FT‐EXAFS in R‐space for Mn metal foil, MnO, NGA‐Mn pure and in the presence of *Escherichia coli* (NGA‐Mn+*E. coli*). f) DFT calculation showing the preferential coordination site of Mn^2+^ with NGA (binding distances are shown in Å, and binding energies in kcal mol^−1^). Carbon – dark gray; hydrogen – white; nitrogen – blue; oxygen – red; manganese – purple.

Here we employed densely and dual‐functionalized graphene with both out‐of‐plane groups and in‐plane heteroatoms (nitrogen‐doped graphene acid, NGA) as a biocompatible and very efficient covalent trap for manganese cations (**Figure** **1**a). Neither NGA nor the manganese ions exert any antibacterial effect when applied independently, but after the immobilization of Mn^2+^ SAs on the graphene, the NGA‐Mn system exhibited potent antibacterial properties against a broad spectrum of multidrug‐resistant bacteria (MIC_100_ between 4–16 mg L^−1^ depending on the strain), while maintaining excellent cytocompatibility (150 mg L^−1^). NGA‐Mn is endowed with unique features: i) it exhibits strong antibacterial activity in vivo and in vitro, similar to that of Ag NPs, and other potent molecular antibiotics^[^ ^8^, ^17^ ^]^ (Tables S5,S6, Supporting Information), even against multidrug‐resistant bacterial strains), ii) it evades bacterial resistance development, even after 30 serial bacterial generation passages, whereas Ag NPs develop resistance at the 9th incubation cycle against *Escherichia coli*, and iii) it has very high cytocompatibility with healthy human cells (ca. 25‐fold higher than the MIC_100_), which is essential for considering its further practical application. NGA‐Mn blocks vital bacterial functions via collective binding to biomolecules on the outer membrane. The current findings reveal a previously untapped and overlooked pathway, which demonstrates how essential micronutrient metal ions can acquire potent antibiotic properties through SAE. This knowledge opens new avenues in combating bacterial resistance.

## 2 Results and Discussion

### 2.1 Single Atom Antibacterial: Synthesis and Structure

The development of the NGA‐Mn antibacterial involved 3 steps: the synthesis of nitrogen‐doped graphene (NG), its oxidation to NGA,^[^ ^57^ ^]^ and the coordination of Mn^2+^ ions on NGA (Figure 1a, and “Methods”). Briefly, fluorographene (FG) reacted with sodium azide to yield NG and then with nitric acid to afford the NGA.^[^ ^57^ ^]^ X‐ray photoelectron spectroscopy (XPS) indicated extensive defluorination and nitrogen doping upon the transformation of FG to NG. Upon transformation of NG to NGA, an increase in oxygen‐containing residues (as carboxyls predominantly) and a decrease in nitrogen content took place (Figure 1b; Figure S1a,b, and Table S1, Supporting Information). These structural features were confirmed by Fourier‑transformed infra‑red spectroscopy (FTIR; Figure 1c; Figure S2, Supporting Information and comments in the caption). Immobilization of Mn^2+^ on NGA resulted in a loading of 4.5 wt.% according to inductively coupled plasma mass spectrometry. The oxidation state of Mn^2+^ after coordination remained the same, as determined by the energy difference in the multiplet splitting in the 3s region by 6.5 eV (Figure S1c).^[^ ^58^ ^]^ The carboxylic groups that were detected by FTIR were partially ionized to carboxylates after the interactions with Mn^2+^, as indicated by the emergence of the vibrations at 1600 cm^−1^ and at 1420/1345 cm^−1^ in NGA‐Mn (Figure 1c; Figure S2a,b, Supporting Information and comments).

We further probed the NGA‐Mn interactions with continuous‐wave electron paramagnetic resonance spectroscopy (CW‐EPR), since both Mn^2+^ and NGA are spin‐active.^[^ ^57^ ^]^ NGA in water (*T* = 80 K, Figure S3a, Supporting Information) shows isotropic and narrow derivative resonance signal (ΔB_pp_ = 0.9 mT), centered at *g*_iso_ = 1.998, which is associated with *S* = 1/2 spin centers that are localized onto the aromatic‐rich regions of the carbon framework (type C‐sp^2^, = C^•^‐) and to spin containing defects placed near/on the carboxyl groups (O = C^•^‐).^[^ ^57^ ^]^ The Mn(NO_3_)_2_ salt dissolved in water displays broad and featureless resonance signal, with a peak‐to‐peak line width of about 77.6 mT at *g* = 2.018 (Figure S3b, Supporting Information). Upon addition of Mn^2+^ ([Ar] 3d^5^) at 10 ppm and 10 ppb to the NGA‐water dispersion, it is observed a clear decrease of the NGA's radical signal intensity (Figure 1d; Figure S3c); the phenomenon results from the electronic interaction, and thus binding of the Mn^2+^ ions^[^ ^59^ ^]^ with the NGA scaffold. The estimated ^55^Mn nuclear hyperfine coupling from EPR simulation (A_iso_ = 9.8 mT) is in harmony with those observed in hexacoordinate Mn^2+^ enzymes, containing a set of N, O donors in distorted octahedral geometries (Figure S4, Supporting Information and comments in the caption).

The coordination of Mn^2+^ cations with light atoms (nitrogen and oxygen) from NGA's functionalities and their single atomic state were verified due to the absence of scattering at radial distances above 2 Å (corresponding to metallic Mn‐Mn or oxide Mn‐O‐Mn bonds), as evidenced by the extended X‐ray absorption near edge (XANES) and extended X‐ray absorption fine structure spectroscopy results (EXAFS, Figure 1e; Table S4 and Figure S5, Supporting Information). These features were preserved in the case of the NGA‐Mn interacting with bacteria (NGA‐Mn+*E. coli*, Figure 1e), as later discussed in more detail. In agreement with these observations, density functional theory (DFT) calculations (Figure 1f) revealed the coordination bonds between Mn2+, the –COOH groups, and nitrogen dopants in NGA according to the natural bond orbital analysis (Tables S2 and S3, Supporting Information). The role of N‐doped vacancies in the coordination of Mn^2+^ was also indicated by the second‐order perturbation analysis which estimated the strength of donor‐acceptor interactions, showing a stronger donation to the vacant orbitals of the metal ions from N compared to the carboxylic oxygens (Tables S2 and S3, Supporting Information). X‐ray diffraction (XRD) also confirmed the absence of any reflections originating from Mn‐based inorganic phases, corroborating the single atomic state of the manganese ions in NGA‐Mn (Figure S6a, Supporting Information).

**Figure 2:**
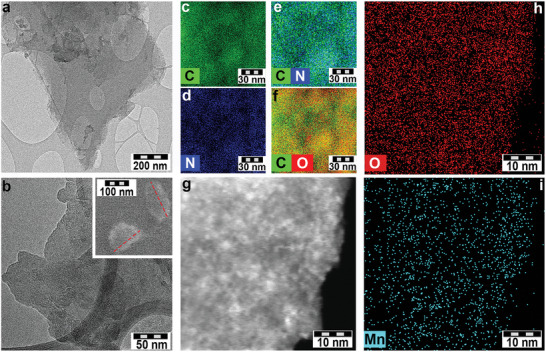
HR‐TEM images of a) NG and b) NGA (inset: SEM image of NGA). EDS chemical mapping of NGA for c) carbon, d) nitrogen, and combined chemical mapping of e) carbon and nitrogen, f) carbon and oxygen. g) HAADF‐STEM image of an NGA‐Mn flake. EDS chemical mapping of NGA‐Mn for h) oxygen and i) manganese.

The analysis of NG, NGA, and NGA‐Mn by high‐resolution transmission electron microscopy (HR‐TEM) and scanning electron microscopy (SEM) showed that NG comprised few‐layered graphene flakes with size ≈600 nm (**Figure** **2**a; Figure S6b,c, Supporting Information), while NGA flakes were smaller, ca. 150 nm, due to the oxidative cutting during its synthesis (Figure 2b, and SEM image with lateral size in the inset). Elemental chemical mapping of NGA confirmed the homogeneous coverage of the flakes with nitrogen and oxygen, coinciding with the spatial distribution of carbon (Figure 2c–f). After the interaction of NGA with Mn^2+^ ions, no Mn‐based NPs were observed on the NGA surface, as confirmed by high‐angle annular dark‐field scanning transmission electron microscopy (HAADF‑STEM, Figure 2g), corroborating the EXAFS results. Elemental chemical mapping (Figure 2h,i) revealed a homogeneous coverage of graphene flakes with Mn cations, as well as oxygen atoms from the carboxyl groups of NGA.

### 2.2 Antibacterial Properties

**Figure 3:**
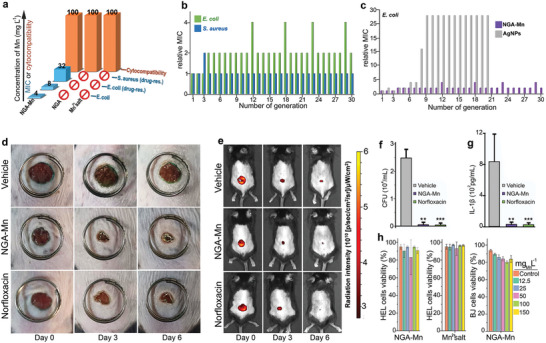
a) MIC values (against *Escherichia coli* CCM 3954, MDR *Escherichia coli* CE 5556, and MDR *Staphylococcus aureus* CCM 4223) and cytocompatibility values (for human lung fibroblasts HEL 12 469) of NGA‐Mn. The concentration of bare NGA (2200 mg L^−1^) for cytocompatibility measurements corresponds to the concentration of Mn (100 mg_Mn_ L^−1^) in the NGA‐Mn composite. In Table S5 (Supporting Information), MIC values with respect to the total NGA‐Mn mass are also available. MIC values were determined according to the European Committee on Antimicrobial Susceptibility Testing,^[^ ^70^ ^]^ as described in the section “Methods”. b) *Escherichia coli* and *Staphylococcus aureus* treated for 30 generations (serial passages) with the NGA‐Mn. c) *Escherichia coli* treated for 30 generations with the NGA‐Mn and for 20 generations with colloidal Ag NPs (mean size 28 nm). Development of resistance is reflected in the changes in relative MIC (defined as the ratio of MIC at generation *n* / MIC at generation 0). The serial passages with colloidal Ag NPs were performed in the frame of a previous publication from some of the authors of this work;^[^ ^38^ ^]^ here the same data are plotted for the sake of comparison. d) Representative optical images illustrating the healing progress of mouse skin wounds upon treatment with vehicle solution (saline), NGA‐Mn, or norfloxacin. e) Representative IVIS fluorescence images depicting fluorescently labeled bacteria on skin wounds. f) Quantification of bacterial colony forming units (CFU) derived from the IVIS fluorescence imaging. Mice with skin wounds were infected with Cy5.5 labeled *Staphylococcus aureus* and subsequently treated topically with either a vehicle solution (saline), 4 mg kg^−1^ NGA‐Mn (equivalent to 0.18 mg kg^−1^ of Mn), or 4 mg kg^−1^ norfloxacin. The growth of bacteria on skin wounds was monitored by the fluorescence of Cy5.5 using an IVIS imaging system (Lumina III, PerkinElmer, USA) every 3 days. Residual bacterial cells were collected from the skin wounds for CFU counting on the 6th day post‐treatment (*n* = 3). g) Concentration of pro‐inflammatory cytokines in washing fluids from skin wounds, verifying the healing process for the NGA‐Mn and norfloxacin. h) Viability of human lung fibroblasts HEL (*n* = 3), human skin fibroblasts BJ (*n* = 3) treated with NGA‐Mn and Mn(NO_3_)_2_. One‐way ANOVA with Dunnett's post hoc test was applied for statistical significance.

With the focus on addressing the alarming implications of bacterial resistance,^[^ ^5^ ^]^ NGA‐Mn was evaluated against Gram‐positive and Gram‐negative antibiotic‐susceptible bacteria, as well as against a wide range of bacteria that are highly resistant to antibiotics such as methicillin‐resistant *Staphylococcus aureus*, vancomycin‐resistant *Enterococcus faecium*, multidrug‐resistant *Escherichia coli* and *Pseudomonas aeruginosa*, extended‐spectrum beta‐lactamase or carbapenemase‐producing *Enterobacter cloacae* (AmpC beta‐lactamase), *Klebsiella pneumoniae* (KPC‐3 carbapenemase), *Klebsiella pneumoniae* (NDM‐1), *Acinetobacter baumannii* (OXA‐23, OXA‐51‐like), and colistin‐resistant *Escherichia coli* (mcr‐1); see “Methods” for detailed description of the bacterial strains. The MIC_100_ ranged at ultra‑low levels, from 4–16 mg_Mn_ L^−1^, (or 90–355 mg L^−1^ with respect to the total NGA‐Mn mass, **Figure** **3**a; Table S5, Supporting Information), while pure NGA and Mn^2+^ salt did not show any antibacterial activity at concentrations as high as 1500 mg L^−1^ (Table S5, Supporting Information). Interestingly, the MIC_100_ values of NGA‐Mn are similar or even lower than those of state‐of‐the‐art antibiotics (**Table** **1**) while, as later discussed, being persistent against bacterial resistance. For example, macolacin^[^ ^8^ ^]^ (a last‐line antibiotic against many Gram‐negative pathogens, but inactive on resistant strains), ETX0462^[^ ^17^ ^]^ (a synthetic potent molecular antibiotic but ineffective against Gram‐positive strains having a thick peptidoglycan outer cell wall), or cilagicin^[^ ^10^ ^]^ (another potent naturally‐inspired lipopeptide antibiotic, ineffective against the double‐membrane Gram‐negative bacteria). Moreover, time‐dependent bactericidal activity showed that NGA‐Mn fully eradicates *Escherichia coli* population after 1 h of incubation and after 12 h for *Staphylococcus aureus* (Figure S7, Supporting Information).

**Table 1:** MIC_100_ (mg L^−1^) values of NGA‐Mn (with respect to Mn) and other antibiotics tested against the ESKAPE pathogens. NGA‐Mn remains effective against all bacterial strains while being persistent against bacterial resistance development, as discussed in section 2.3.

	Bacterial strain	Macolacin^[^ ^8^ ^]^	ETX0462^[^ ^17^ ^]^	Cilagicin^[^ ^10^ ^]^	NGA‐Mn (this work)
*E*	*Enterococcus faecium‐ resistant*	>125	ineffective	1	16
*S*	*Staphylococcus aureus – resistant*	>125	ineffective	1	16
*K*	*Klebsiella pneumoniae*	1	4 (MIC_90_)	>64	16
*A*	*Acinetobacter baumannii*	1	4 (MIC_90_)	8	4
*P*	*Pseudomonas aeruginosa*	4	4	>64	4
*E*	*Enterobacter cloacae*	4	NA	>64	4

### 2.3 Evaluation of Bacterial Resistance

Microorganisms have developed sophisticated defense mechanisms over the billion years of their natural evolution against potential threats, even in the case of silver ions and Ag NPs.^[^ ^20^, ^21^, ^38^ ^]^ Thus, in order to establish the persistence of the antibacterial activity of NGA‐Mn, serial passages^[^ ^60^ ^]^ were performed for 30 generations of *Escherichia coli* and *Staphylococcus aureus*. (Figure 3b). The MIC_100_ of NGA‐Mn increased marginally in the case of *Escherichia coli*, and no changes were observed for *Staphylococcus aureus* (Figure 3b). An eightfold increase in the MIC value is considered indicative of resistance development.^[^ ^38^ ^]^ The absence of resistance development suggests that the NGA‐Mn's target is not a protein.^[^ ^61^ ^]^ For comparison, after exposing *Escherichia coli* to Ag NPs, resistance was developed at the 9th cycle, indicated by the twenty‐eight‐fold increase in MIC (Figure 3c). These findings suggest NGA‐Mn as a next‐generation, persistent, and broad‐spectrum antibacterial agent, with a combination of features that are challenging to achieve.^[^ ^7^, ^8^, ^11^, ^16^, ^17^ ^]^ Moreover, the high antibacterial activity of NGA‐Mn against multidrug‐resistant bacteria substantially outperforms recently reported nano‐ and SAE systems, since NGA‐Mn exhibits lower MIC values and does not require light irradiation,^[^ ^45^, ^56^, ^62^ ^]^ ultrasonic action^[^ ^63^, ^64^ ^]^ or co‐presence of additional agents^[^ ^52^, ^53^, ^54^, ^65^ ^]^ to generate reactive oxygen species (Table S6, Supporting Information). In Table S6 (Supporting Information), the activity of NGA‐Mn is also contextualized with respect to potent molecular antibiotics, revealing the unique combination of broad‐spectrum activity with persistence against bacterial resistance development.

Building on the promising in vitro results, the antibacterial activity of NGA‐Mn was evaluated in vivo in a mouse skin wound model infected with penicillin‐resistant *Staphylococcus aureus*. The application of NGA‐Mn at a dosage of 4 mg kg^−1^ (equivalent to 0.18 mg kg^−1^ of Mn) facilitated remarkably efficient wound healing (Figure 3d–g; Figure S8, Supporting Information). The healing was similar or improved than that achieved with the commercial antibiotic of norfloxacin (at a dose of 4 mg kg^−1^, Figure 3d–g; Figure S8, Supporting Information), a broad‐spectrum antibiotic with activity against both Gram‐positive and Gram‐negative bacteria, but limited due to bacterial resistance development.

### 2.4 Acute and Long‐Term Cytocompatibility

An equally important aspect of antibacterial agents is their safety. The cytocompatibility of NGA‐Mn was investigated with flow cytometry (using propidium iodide and calcein fluorescent probes, see “Methods”) on human skin fibroblasts because of the potential application of antibacterial agents on the skin and on human lung fibroblasts (HEL 12 469) for further establishment of the cytocompatibility profile. In addition, the THP‐1 cell line, a model for immune‐competent cells^[^ ^66^ ^]^ was also evaluated as a crucial aspect of safety assessment, considering potential immunotoxicity. It was very gratifying to observe that NGA‐Mn was fully tolerated by all cell lines up to 2200 mg L^−1^ (or 100 mg_Mn_ L^−1^, Figure 3h; Figure S9, Supporting Information) for 24 h, which is ca. 3‐ to 25‐fold higher (depending on the bacterial strain) than its antibacterial MIC values (Figure 3a). The same cytocompatibility results were obtained when tests were performed in a culture medium without proteins, in order to secure that the cytocompatibility is not due to protein corona formation^[^ ^67^ ^]^ (Figure S10, Supporting Information). The longer‐term safety profile of NGA‐Mn was also investigated for 72 h (Supporting Information section “Methods‐Long term safety assessment”), confirming the exceptional biocompatibility of NGA‐Mn, as the viability exceeded 80% for all tested concentrations (Figure S11, Supporting Information). Repeated exposure of cells to NGA‐Mn (10 mg_Mn_ L^−1^ total dose) for 14 days was also implemented (Figure S12, Supporting Information). On day 15, 3 different endpoints (cell viability, cell cycle, and cell metabolic activity) were evaluated to comprehensively capture the biological response of the cell models to NGA‐Mn. The data demonstrated that the proliferation rates were the same, with no significant differences in cell viability, cell cycle profile, and cell metabolic activity, between NGA‐Mn treated and untreated cells (Figure S13, Supporting Information).

Manganese leaching tests from the NGA‐Mn confirmed the experiments and simulations about the strong binding of Mn, since after 72 h of shaking in water or in a cell‐culture medium, Mn leaching reached 0.15 mg L^−1^ (Figure S14, Supporting Information), well below the toxic levels of NGA‐Mn or Mn^2+^ salt (150 mg_Mn_ L^−1^, Figure 3d). The leached amount of Mn corresponded to 0.05% of the total amount of Mn originally contained in NGA‐Mn that was added to the leaching test solution. These results underscore the application potential of the NGA‐Mn antibiotic, particularly considering the general cytotoxic effect of silver‐based agents^[^ ^68^ ^]^ or the yet unknown cytocompatibility of other state‐of‐the‐art SAE antibacterials.^[^ ^45^, ^53^, ^62^, ^64^, ^69^ ^]^ Such high cytocompatibility, combined with the potent and persistent antibacterial activity of NGA‐Mn against multidrug‐resistant bacteria, may provide a new thrust in the battle against superbugs.

### 2.5 Mechanism of Antibacterial Activity

**Figure 4:**
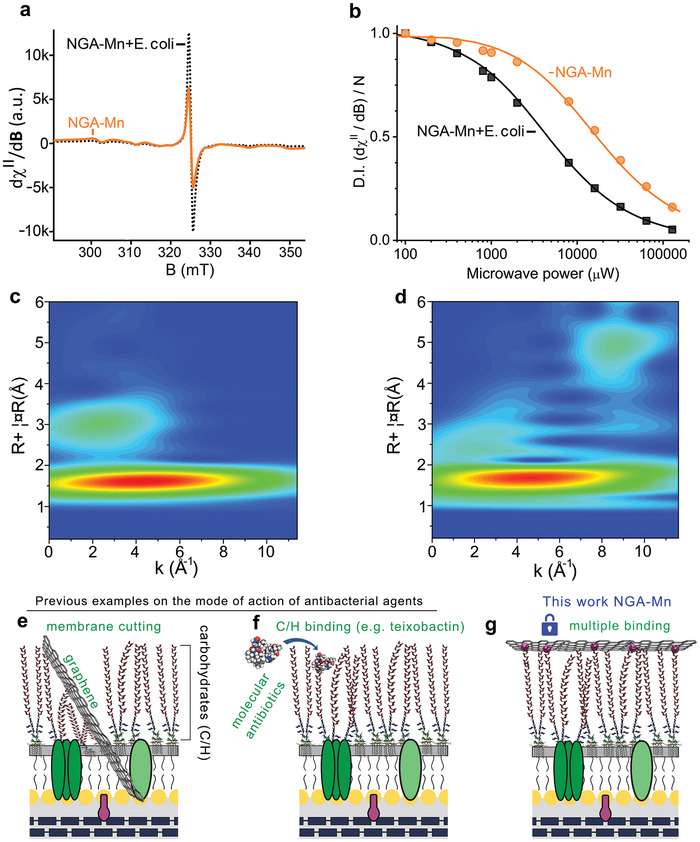
a) EPR spectra of NGA‐Mn in the bacterial medium before and after addition of *Escherichia coli* (NGA‐Mn+*Escherichia coli*). b) The microwave power saturation trends of the EPR signal of NGA‐Mn in growth medium before and after the addition of Escherichia coli +, The solid lines represent the theoretical simulation of the saturation profiles. c) WT‐EXAFS for NGA‐Mn, and d) for NGA‐Mn+*Escherichia coli*. Representative examples of the mode of action of antibacterial agents e) mechanical membrane cutting by graphene/graphene oxide,^[^ ^72^ ^]^ f) specific binding of molecular antibiotics to biomolecules (carbohydrates C/H). In the particular example, we selected teixobactin, a newly discovered natural oligopeptide that binds the C/H (teichoic acids) on the outer membrane of Gram‐positive bacteria,^[^ ^71^ ^]^ and g) multiple binding of the numerous immobilized Mn^2+^ ions (purple spheres) on one NGA sheet to several carbohydrate residues on the outer bacterial membrane. Note: The scheme depicts part of the outer membrane of Gram‐negative bacteria whose ECM contains a wall of lipopolysaccharides. In the case of panel “f” regarding teixobactin, which is active only on Gram‐positive bacteria, the ECM is made from a dense network of polysaccharides called teichoic acid. For simplicity, with have used the same model of Gram‐negative bacteria.

To gain insight into the mode of antibacterial activity, we studied the NGA‐Mn/bacteria interactions combining EPR and EXAFS. Initially, the coordination of Mn^2+^ cations to neat NGA led to NGA's EPR signal decrease (Figure 1d). However, the subsequent addition of NGA‐Mn to *Escherichia coli* partially reversed the process (**Figure** **4**a), which is indicative of a modification of the coordination environment of Mn^2+^ in NGA due to the newly developed interactions between NGA‐Mn with the bacterial biomolecules. The coordination of Mn to bacteria was further probed by recording the power saturation behavior of the EPR resonance signals of NGA‐Mn in the absence and in the presence of *Escherichia coli* (Figure 4b). The Mn^2+^ signal saturation behavior significantly changed upon the addition of *Escherichia coli*, showing a large decrease in the P_1/2_ value (the power at which the signal is half‐saturated), indicating the increased disorder (longer spin‐lattice relaxation, T1) in the Mn ligand‐field arising from multiple binding motives of Mn^2+^ to bacteria. The power saturation results also suggest the absence of released Mn cations from NGA‐Mn into the bulk upon interaction with the bacteria (see comments in Supporting information page 23). In all cases, EPR signals were recorded immediately after the addition of bacteria and freezing. The FT‐EXAFS (Figure 1e) and wavelet transformed EXAFS (WT‐EXAFS, Figure 4b,c) verified that in the presence of bacteria, Mn retained the coordination pattern with NGA and the atomic distribution of Mn ions, with altered scattering profile only at high radial distances (>2 Å) beyond the first coordination sphere (particularly at ca. 5 Å, and 9 Å^−1^). This reflects the complex broader coordination environment imposed by the attached bacterial biomolecules. Only slight changes in the first coordination sphere are observed (at radial distances below 2 Å), since the light atoms of the biomolecules do not differentiate in the XANES/EXAFS. Overall, both EPR and EXAFS demonstrate the coordination of the Mn on NGA and on bacteria.

Coarse‐grained molecular dynamics simulations in explicit solvent showed that NGA and NGA‐Mn attach to *Escherichia coli* membrane, with NGA‐Mn having a higher affinity to the lipid membrane (Figure S15–S18, Supporting Information). No penetration into the hydrophobic membrane interior or mechanical disruption of the membrane was observed on the simulation timescales after 6 µs (Figure S15, Supporting Information). Similar results from simulations were obtained with other divalent and trivalent cations, which did not exert significant antibacterial activity (Figure S19, Supporting Information), supporting that NGA‐Mn's antibacterial action is rather not related to electrostatic interactions (on which these models are based) or to membrane cutting. The latter is in agreement with the absence of any Mn transfer inside the cells, according to trace analysis of bacteria treated with NGA‐Mn. SEM imaging of the bacteria before and after incubation with NGA (Figure S20a–c, Supporting Information) and NGA‐Mn (Figure S20d–h, Supporting Information) showed alterations on the bacterial membrane only in the case of NGA‐Mn. Similar pits on the membrane were observed also on multi‐resistant *Staphylococcus aureus* (FigureS20i–m, Supporting Information). Laser scanning confocal microscopy (Figure S20n–p, Supporting Information) with dual green and red staining was used to distinguish between bacteria with intact (Figure S20n, Supporting Information) and disrupted (Figure S20o, Supporting Information) membranes, respectively, also validating that membrane disruption only occurs in the case of NGA‐Mn (Figure S20p, Supporting Information). The observed membrane perturbation should be the consequence of NGA‐Mn‐binding on the extracellular matrix (ECM), impairing vital functions of the bound biomolecules. The outer parts of ECM are composed of biomolecules with saccharide‐specific sequences (e.g., teichoic acids in Gram‐positive and lipopolysaccharides in Gram‐negative bacteria), and they are involved in many vital processes, such as in membrane synthesis,^[^ ^6^ ^]^ which in the case of eukaryotic cells are implemented by specialized intracellular organelles that are absent in bacteria. Thus, concerted efforts focus on extracellular wall targets in order to avoid the inner bacterial membrane ejection pumps.^[^ ^6^, ^71^ ^]^

The accumulated data suggest the possible mechanism of NGA‐Mn function: i) since neither the application of free Mn^2+^ cations nor free NGA exert any antibacterial effect, ii) the NGA‐Mn does not penetrate the cell membrane, and since iii) NGA‐Mn binds the bacteria immediately after their mixing, it is evident that the antibacterial action arises from the collective binding of the numerous immobilized Mn^2+^ ions on the 150 nm‐NGA sheets to multiple outer membrane carbohydrate biomolecules. This was further supported by control experiments where the antibacterial activity (expressed as MIC in mg of Mn per liter) was substantially compromised when an NGA‐Mn system was tested with lower Mn content. This outcome underscores the critical role of the spatial density of Mn ions on the NGA surface in facilitating multiple and simultaneous binding events, essential for preserving its antibacterial efficacy (see comment in Supporting Information, page 18). Such intricate binding can severely restrict cell‐wall synthesis,^[^ ^6^ ^]^ for which ECM carbohydrates are responsible. The absence of reactive oxygen species generation during NGA‐Mn interaction with bacteria (Figure S21, Supporting Information) also excludes other antibacterial action through the production of cytotoxic radical species, which would be toxic for human cells as well. Certainly, further research is necessary to determine specifically the oligosaccharide that NGA‐Mn binds to. The multiple and bridging binding of biomolecules by the NGA‐Mn agent introduces a striking departure from the conventional mechanical membrane disruption by graphene sheets (Figure 4e), or antibiotics binding to one biomolecule (Figure 4f). The antibacterial activity of NGA‐Mn via collective binding (Figure 4g) and its persistence for 30 bacterial generations (the maximum tested in this study), opens the door to the discovery of new tools based on SAE for evading drug resistance development.

## 3 Conclusion

The outbreak of antibiotic‐resistant bacteria, or “superbugs,” threatening global health necessitates the urgent development of potent antimicrobial agents that remain impervious to bacterial resistance development. Despite intense efforts during the last decades, only recently, teixobactin was reported to be active and persistent against Gram‐positive only bacteria, acting through the simultaneous binding of 2 extracellular hydrocarbon bacterial targets. The herein study contributes to this endeavor by introducing a previously overlooked strategy for the development of potent, persistent (i.e., bypassing bacterial resistance development), and broad‐spectrum antibacterial agents via single‐atom engineering without the use of any molecular antibiotic. In particular, the strong coordination of manganese cations on a functionalized graphene derivative (termed NGA‐Mn) totally inhibits the growth of both Gram‐positive and Gram‐negative multi‐drug resistant bacteria. This is experimentally verified although both the functionalized NGA graphene and the manganese cations are cytocompatible and non‐bactericidal when applied independently. Most importantly, NGA‐Mn is not only a potent in vitro and in vivo antimicrobial, but it is also persistent, as demonstrated by its effectiveness during serial passages over thirty bacterial generations, emphasizing its ability to bypass bacterial resistance mechanisms. Another key feature is the high cytocompatibility toward human cells, rendering the toxicity of NGA‐Mn selective only against bacterial strains. Experimental evidence suggests that such unprecedented activity can be attributed to the simultaneous binding of NGA‐Mn in bridging and rigid configuration to multiple bacterial extracellular biomolecules, whose mobility is critical for membrane synthesis and survival of the bacteria. Given the similarity of the suggested activity mechanism with the recently discovered mechanism of teixobactin, the present findings might dictate an effective and general strategy in the battle against bacterial resistance. Although further studies are required to understand the function of NGA‐Mn, the importance of this work is understood considering the limited examples of effective antibiotics that have been discovered during the last 25 to 50 years.^[^ ^7^, ^17^ ^]^ Thus, the work conveys a promising finding in the field, paving the way toward further developments urgently required for winning the battle against superbugs and their multidrug resistance.

## 4 Experimental Section

### Cell Cultures

For cellular toxicity, human lung fibroblasts HEL 12 469, human skin fibroblasts BJ (both ATCC), and human monocyte‐like THP‐1 (ECOCC) cell lines were used. HEL and BJ cells were cultivated at 37 °C under a 5% CO_2_ atmosphere in EMEM—Eagle's Minimum Essential Medium (Sigma‐Aldrich), enriched with L‐Glutamine, non‐essential amino acids (NEAA), fetal bovine serum (FBS), PenStrep (5000 U penicillin, 5 mg streptomycin mL^−1^), and sodium bicarbonate (7.5%). The THP‐1 cells were cultivated at 37 °C under a 5% CO_2_ atmosphere in RPMI‐1640 medium (Sigma‐Aldrich) supplemented with 1% L‐Glutamine, 10% FBS, and 1% PenStrep (10 000 U penicillin, 10 mg streptomycin mL^−1^). Leaching tests were conducted in EMEM M5650 which contains 21 aminoacids and several salts (0.265 g L^−1^ CaCl_2_, 0.097 g L^−1^MgSO_4_, 0.4 g L^−1^KCl, 0.122 g L^−1^NaH_2_PO_4_, 2.2 g L^−1^NaHCO_3_, 6.8 g L^−1^NaCl), aiming at closely mimicing the conditions found in living organisms and to evaluate the stability of manganese in the presence of potential competing cations and substances.

### Cell Viability Assay

Cell viability was evaluated using a BD FACS Verse flow cytometer (BD Biosciences). Ten thousand cells were seeded per well in a 96‐well plate. Cells were incubated with NGA‐Mn at various concentrations (based on the total mass of Mn) from 12.5 to 150 mg L^−1^ (for HEL and BJ cells) and 12.5 to 100 mg L^−1^ (for THP‐1 cells) for 24 h. Prolonged incubation with 12.5 to 100 mg L^−1^ of NGA‐Mn (based on the total mass of Mn) for 72 h in all 3 cell models was also performed, where cells were seeded in 24‐well plate (50 000 cells/well). In addition, to determine the role of protein corona in the acute toxicity of NGA‐Mn, the cell viability was also assessed in a culture medium without the supplementation of 10% FBS. All 3 cell lines were again treated with various concentrations of NGA‐Mn (based on the total mass of Mn) from 12.5 to 100 mg L^−1^ for 24 h. For these experiments, the supernatant was collected, and cells were gently washed with PBS solution (0.1 M, pH 7.4) after the treatment. Then, cells were detached with trypsin (0.25% in EDTA, Sigma‐Aldrich), resuspended in 100 µL of culture media, and added to the supernatant. The viability of cells was determined by propidium iodide (PI) and calcein‐AM fluorescent probes. Cells were incubated with 1 µL of PI (1 µg mL^−1^) and 2 µL of calcein‐AM, diluted in DMSO (50 µM), for 20 min and the fluorescent signal was measured by flow cytometer using a red channel (exc. 488/em. 586) for PI and green channel (exc. 488/em. 527) for calcein. The red signal of PI revealed dead cells, which lost their membrane integrity, while the green signal was represented by cells with active intracellular esterases that catalyzed the non‐fluorescent calcein‐AM to highly fluorescent green calcein.

### Cell Cycle Analysis

Cell cycle analysis was performed using BD Cycletest Plus DNA kit (BD Biosciences). BJ and HEL cells were seeded in 96‐well plates (10^4^ cells per well), while THP‐1 cells were seeded in 24‐well plates (5 × 10^4^ cells per well). The next day, cells were treated with various concentrations of NGA‐Mn (based on the total mass of Mn) or NGA alone (equal to the mass of Mn in NGA‐Mn) from 12.5 to 100 mg L^−1^ for 24 h. Then, the supernatant was removed, cells were washed with warm PBS (0.1 M, pH 7.4), and kit solutions were added according to the manufacturer's protocol. For the THP‐1 cells, a centrifugation step (1500 rpm, 5 min) was implemented for supernatant removal and PBS washing. Finally, fluorescence was measured on BD FACS Verse flow cytometer (BD Biosciences).

### Long‐Term Safety Assessment

For the assessment of the long‐term safety of NGA‐Mn, the adjusted protocol from Mukherjee et al.^[^ ^73^ ^]^ was used. Briefly, THP‐1, HEL, and BJ cells were seeded in T25 flasks (1.25 × 10^5^ per flask) and cumulatively treated 4 times with 2.5 mg L^−1^ of NGA‐Mn (based on the total mass of Mn) after various time points for 14 days (cumulative dose 10 mg L^−1^ of NGA‐Mn (based on the total mass of Mn)). On day 14, cells were harvested, re‐seeded in 96‐well plates (10^4^ cells per well) in complete culture medium without NGA‐Mn, and incubated for 24 h. Then, on day 15, the analysis of cell cycle, cell viability, and cell metabolic activity was performed. For the detailed scheme of the experiment, please refer to Figure S12a. Cell cycle and cell viability were assessed as described above. For the cell metabolic activity, Alamar blue assay (Thermo Fisher Scientific) was used. After treatment, the culture medium was replaced with 10% Alamar blue solution (Thermo Fisher Scientific) in cell culture medium (for THP‐1 cells, samples were centrifuged (1500 rpm, 5 min) due to the suspension nature of the cell line) and cells were incubated for 2 h in the cell incubator (37 °C, 5% CO_2_). Then, fluorescence was measured (ex. 560/em. 590 nm) using the Infinite PRO M200 microplate reader (Tecan, Austria).

### Animal Experiments

Six‐week‐old C57BL/6 mice obtained from Peng Sheng Biological Technology (Nanjing, Jiangsu, China) were used to establish the animal infection model. The mice were housed under standard laboratory conditions (20–26 °C, 40%–70% humidity, 12 h light, and 12 h dark), and received autoclaved food and acidified water. Animal treatment protocols were approved by the Soochow University Laboratory Animal Center. Mice were anesthetized by intraperitoneal injection of sodium pentobarbital (200 mg kg^−1^) in a total volume of 100 µL. A circular wound of 6 mm in diameter was created over the dorsal skin area of each mouse after the removal of the hair. Wounds were infected with Cy5.5 labeled S. aureus (≈2 × 10^7^ CFU per mouse). After 6 h, the infected animals were randomly divided into 3 groups and received topical treatments with a vehicle solution (20 µL saline), 4 mg kg^−1^ graphene (20 µL, 5 mg mL^−1^), or 4 mg kg^−1^ norfloxacin (20 µL, 5 mg mL^−1^).

Labeled bacteria on wounds were visualized using a Lumina III IVIS imaging system (PerkinElmer, USA) every 3 days.^[^ ^74^ ^]^ Photographs of skin wounds were captured using a Canon camera. On the 6th day, skin wounds were washed with 1 mL PBS for CFU counting^[^ ^75^ ^]^ and detection of pro‐inflammatory cytokines, including IL‐1β and TNF‐α by commercial ELISA kits (BD Biosciences, NJ, USA). Healed skins were collected on the 9th day for histological examination using H&E staining. The animal experiments were performed in accordance with the guidelines approved by the Animal Care Committee of the Laboratory Animals at Soochow University (No. 202401A0333).

### Statistical Analysis

In the acute and long‐term safety cytocompatibility assays, 3 experiments were performed at least in duplicate. For the Alamar blue assay (metabolic activity) of long‐term cytocompatibility evaluation, data were normalized for 100% of cell metabolic activity based on the untreated control. Data are represented as mean values ± S.D., in either standard column charts or charts displaying all the individual points. Statistics (*n* = 3) were performed with one‐way ANOVA with Dunnett post hoc test versus control or Student's t‐test of treated sample versus control using Statistica software. The statistical significance was highlighted with asterisks: *p ≤ 0.05, **p ≤ 0.01, ***p ≤ 0.001. All animal experiments were repeated at least thrice with 3 to 10 replicates. Data were expressed as mean ± standard deviation (SD) from at least 3 replicates. All confocal imaging and immunohistochemical staining imaging were repeated with at least 3 replicates. Data analysis was performed by a two‐tailed Student's t‐test or Log‐rank test via SPSS Statistics 17.0 software. The difference was regarded as statistically significant if *p* < 0.05.

## Conflict of Interest

The authors declare no conflict of interest.

## Author Contributions

D.P., J.B., L.H., Z.B., G.Z., V.Š., R.V., P.K., J.Z., L.L., E.Z., Z.C., M.Š., M.P., R.L., L.Z., T.M., Z.X. and R.L. performed methodology and investigation. A.B. and R.Z. performed investigation, supervision, wrote, reviewed and edited the original draft. D.P., A.B., Z.B., G.Z., M.P., and R.L. performed Investigation, and visualization. M.K., M.O., R.Z. performed funding acquisition and supervision, wrote, reviewed and edited the original draft. D.P., A.B., G.Z. performed visualization, investigation, wrote original draft. A.P., M.K., M.O., wrote, reviewed and edited the original draft.

## Supporting information



Supporting Information

## Data Availability

The data that support the findings of this study are available from the corresponding author upon reasonable request.
