# Supplementary material for: Single Atom Engineered Antibiotics Overcome Bacterial Resistance
Source: Adv Mater. 2024 Sep 23;36(50):2410652. doi: 10.1002/adma.202410652 (PMC11635910; doi:10.1002/adma.202410652)
Supplement: Supplementary file 1 — Supporting Information [file ADMA-36-2410652-s001.docx]

**Supporting Information**

**Single Atom Engineered Antibiotics Overcome Bacterial Resistance**

*David Panáček^1,2^, Jan Belza^1^, Lucie Hochvaldová^3^, Zdeněk Baďura^1,2^, Giorgio Zoppellaro^1,2^, Martin Šrejber^1^, Tomáš Malina^1,2^, Veronika Šedajová^1^, Markéta Paloncýová^1^, Rostislav Langer^4^, Lukáš Zdražil^1,2^, Jianrong Zeng^5^, Lina Li^5^, En Zhao^6^, Zupeng Chen^6^, Zhiqiang Xiong^7^, Ruibin Li^7^, Aleš Panáček^3^, Renata Večeřová^8^, Pavla Kučová^8^, Milan Kolář^8^, Michal Otyepka^1,4^, Aristides Bakandritsos*^1,2^ and Radek Zbořil*^1,2^*

^1^Regional Centre of Advanced Technologies and Materials, Czech Advanced Technology and Research Institute (CATRIN), Šlechtitelů 241/27, 783 71, Olomouc – Holice, Palacký University Olomouc, Czech Republic

^2^Nanotechnology Centre, Centre for Energy and Environmental Technologies, VŠB–Technical University of Ostrava, 17. listopadu 2172/15, 708 00 Ostrava-Poruba, Czech Republic

^3^Department of Physical Chemistry, Faculty of Science, Palacký University Olomouc, 17. listopadu 1192/12, 771 46 Olomouc, Czech Republic

^4^IT4Innovations, VŠB-Technical University of Ostrava, 17. listopadu 2172/15, 708 00 Ostrava-Poruba, Czech Republic

^5^Shanghai Synchrotron Radiation Facility, Shanghai Advanced Research Institute, Chinese Academy of Sciences, 201204 Shanghai, P. R. China.

^6^Jiangsu Co-Innovation Center of Efficient Processing and Utilization of Forest Resources, International Innovation Center for Forest Chemicals and Materials, College of Chemical Engineering, Nanjing Forestry University, Longpan Road 159, Nanjing 210037, China

^7^State Key Laboratory of Radiation Medicine and Protection, School for Radiological and Interdisciplinary Sciences (RAD-X), Collaborative Innovation Center of Radiation Medicine of Jiangsu Higher Education Institutions, Suzhou Medical College, Soochow University, Suzhou, Jiangsu 215123, China

^8^Department of Microbiology, Faculty of Medicine and Dentistry, Palacký University Olomouc, Hněvotínská 3, 779 00 Olomouc, Czech Republic

**Materials and methods**

**Chemicals and reagents.** Graphite, fluorinated polymer > 61 wt. % F – GF, (Millipore Sigma), sodium azide 99% - NaN_3_ (Millipore Sigma), dimethylformamide pure -DMF (Lach:ner), absolute ethanol – EtOH (Penta), nitric acid 65% - HNO_3_ (Lach:ner), acetone 99% (Millipore Sigma). Aqueous stock solutions of manganese [Mn^2+^], copper [Cu^2+^], nickel [Ni^2+^], and iron [Fe^3+^] were prepared from the nitrate salt (p.a., Merck). Ultrapure water (18 MΩ cm) was used for all solutions.

**Synthesis of nitrogen-doped graphene (NG).** The synthesis of NG was performed according to the previous report.^1^ 10 g of fluorographite was dispersed in a glass flask in 300 ml of DMF stirred for 72 h, and sonicated for 4 h. Then 30 g of NaN_3_ was added to the previous mixture, transferred to spherical glass flask and was stirred and heated at 130°C for 3 days with a condenser. After cooling down, the solid product was washed with DMF (3x), acetone (3x), ethanol (3x), distilled water (3x) and hot distilled water (2x), using centrifugation (14000 rcf).

**Synthesis of carboxyl functionalized nitrogen-doped graphene (NGA).** The synthesis of NGA was performed according to the previous report^2^, but using 45 % of HNO_3_ instead of 65 %. An amount of the previously prepared N-doped graphene derivative was treated with 45% HNO_3_ for 48 hours at 130°C in a glass flask with the condenser. After completion of the reaction, the product was purified by washing with filtration (Whatman cellulose membrane filter, pore size 0.2 μm); 5 times with hot distilled water, 5 times with distilled water and purified by dialysis (dialysis tubing cellulose membrane, 14 kDa cutoff).

For antibacterial applications, after the purification step, the NGA was filtered using a 200 nm cellulose filter in the first step, then the filtrate was taken and filtered again using a 100 nm cellulose filter (Millipore cellulose membrane filter, pore size 0.1 μm). The NGA remaining on the 100 nm filter was used to immobilize the manganese ions.

**Synthesis of NGA-single-atom hybrids.** For this purpose, a 5 mL aqueous suspension containing 25 mg of NGA was mixed with 10 mL of 30×10^–3^ M Mn(NO_3_)_2_ solution and with 10 mL of 1×10^–3^ M Cu(NO_3_)_2_, Ni(NO_3_)_2_ and Fe(NO_3_)_3_ solution and then sonicated for 30 min. Then each mixture was stirred vigorously for 24 h at room temperature, in the dark. The pH during the synthesis was ca. 3.5. The dispersion of the NGA with immobilized single atoms was purified by washing five times with distilled water on a filter paper (Millipore cellulose membrane, pore size 0.1 μm) in order to remove the metal ions not firmly coordinated on NGA. NGA-Mn, NGA-Fe, NGA-Cu, and NGA-Ni were obtained with metal contents of 4.5, 4.1, 4.4 and 4.7 wt.%, respectively. The synthesis of 1.4 wt.% NGA-Mn and 2.6 wt.% NGA-Mn was performed in the same way as the other NGA-metal systems, but using 10 mL solutions of 1x10^-3^ M and 15x10^-3^ M in Mn(NO_3_)_2_, instead of 30×10^–3^ M for the 4.5 wt.% NGA-Mn.

**Synthesis of colloidal silver nanoparticles.** Colloidal silver 28 nm sized particles were synthesized *via* a modified Tollen’s method using maltose as reducing agent, according to Panacek et al.^3^ Briefly, [Ag(NH_3_)^2^]^+^ complex (prepared by mixing AgNO_3_ with NH_4_OH solution) was reacted with maltose at room temperature for 5 min. The initial concentrations of the reaction components were 10^–3^ mol L^–1^ and 0.01 mol L^–1^ for AgNO_3_ and the reducing agent, respectively. The concentration of ammonia was 0.005 mol L^–1^.

**Antibacterial assay.** Antibacterial activity was evaluated by determining MIC (minimum inhibitory concentration) and MBC (minimum bactericidal concentration) using the standard microdilution method according EUCAST (The European Committee on Antimicrobial Susceptibility Testing – EUCAST. http://www.eucast.org). Tested substances were diluted in MH medium (Mueller Hinton broth (MH broth), BIORAD, ref. 69444: Dehydrated beef extract infusion 2 g L^-1^, casein hydrolysate 17.5 g L^-1^, starch 1.5 g L^-1^. Final pH: 7.3 ± 0.1 Ca^2+^ 20-25 mg L^-1^ Mg^2+^ 10-12.5 mg L^-1^) to obtain a concentration range between 256 mg L^-1^ and 2 mg L^-1^. The plates were inoculated with a standard amount of the tested microbe - the initial density of the inoculum was equal to 5 x 10^5^ CFU/ml. MICs were read after 18 ± 2 hours of incubation at 35 ± 1°C. The same procedure with slight modifications was used to determine MIC of yeast tested. The test substances were diluted in Sabouraud broth (BIORAD, peptone 11 g L^-1^, glucose 20g/l, Final pH 6.0 – 6.3) with 2% glucose (BioRad, France). MICs were read after 48 h of incubation at 35 ± 1 °C. The MIC was defined as the lowest concentration of antibacterial with no visible growth. To determine MBC, 10 µl from each well with visibly inhibited growth was inoculated onto MH agar/Sabouraud agar (Trios, Czech Republic) and incubated for another 18 h at 35±1 °C. The MBC is defined as the lowest drug concentration which kills 99.9% of the test microorganisms in the original inoculum. All tests were performed in duplicate.

**Bacterial Strains.** Standard reference strains *Enterococcus faecalis* CCM 4224, *Staphylococcus aureus* CCM 4223, *Staphylococcus epidermidis* CCM 7221, *Escherichia* coli CCM 3954, *Pseudomonas aeruginosa* CCM 3955 and *Candida albicans* CCM 8161 from the Czech Collection of Microorganisms (CCM, Brno, Czech Republic) were tested. Bacterial strains with confirmed antibiotic resistance were also included in the experiment. Methicillin-resistant *Staphylococcus aureus* 4591/A (PBP2a+), vancomycin-resistant *Enterococcus faecium* 419/ANA (VanA), multidrug-resistant *Escherichia coli* CE5556 (CTX-M-15, gyrA, aminoglycoside resistant) and multidrug-resistant *Pseudomonas aeruginosa* (PDC+) strains were obtained from the culture collection of Department of Microbiology, Faculty of Medicine and Dentistry, Palacký University Olomouc, Czech Republic. Strains with confirmed extended-spectrum beta-lactamase or carbapenemase production were obtained from NCTC (UK): *Escherichia coli* NCTC 13353 (CTX-M-15), *Enterobacter cloacae* NCTC 13406 (AmpC beta-lactamase), K*lebsiella pneumoniae* NCTC 13438 (KPC-3 carbapenemase), *Klebsiella pneumoniae* NCTC 13443 (NDM-1), *Acinetobacter baumannii* NCTC 13301 (OXA-23, OXA-51-like). *Escherichia coli* NCTC 13846 was colistin resistant (mcr-1). All tested microorganisms were identified by the MALDI-TOF Biotyper system (Bruker Daltonics, Germany) and stored in cryotubes (ITEST plus, Czech Republic) at -80 °C.

**Induction of resistance.** Induction of bacterial resistance was performed by the dilution micromethod by repeated exposure of *Escherichia coli* CCM 3954 and *Staphylococcus aureus* CCM 4223 strains to sub-inhibitory concentrations of the test compounds of AgNPs and NGA-Mn. The test substances were exponentially diluted in MH culture broth (Mueller Hinton Broth, Biorad, France; the highest concentration was 128 mg L^-1^. The prepared plates were stored at -20°C.

Bacterial cultures were prepared from inoculum in saline and inoculated into a pre-prepared microtiter plate. The initial concentration of the bacterial suspension in the well was approximately 5x10^5^ CFU/ml. After 18 hours of incubation at 35 °C, the MIC value was read. After incubation and MIC reading, 10 µl of the bacterial suspension from the well with the highest sub-inhibitory concentration of the test substance was plated onto blood agar and incubated for 24 hours at 35 °C. The bacterial culture thus obtained was used for the next cycle. The procedure described represents 1 cycle of resistance induction and a total of 30 cycles were performed. After the 10th, 16th, and 30th cycles, the MICs of the original strains were determined and compared with the MICs of the strains after induction.

**Determination of manganese ion concentration in bacteria.** For this purpose, incubation of *Escherichia coli* with NGA-Mn material and manganese salt Mn(NO_3_)_2_ was performed according to the protocol described above (Antibacterial assay). At the end of the incubation (18 h), the solution containing the NGA-Mn with bacteria and culture medium was washed three times with phosphate-buffered saline (PBS) on a 450 nm pore-size cellulose filter to remove all material from the bacteria. Then the same process was carried out with distilled water. The filter containing the bacteria washed this way was placed in 5 ml of distilled water, and the bacteria were transferred to the solution by vigorous shaking. The solution was submitted to scanning electron microscopy to confirm the presence of bacteria in the solution. Nitric acid was then added to the solution containing bacteria to give a final nitric acid concentration of 10 %. Thus, the solution was sonicated for 1 hour to break down the bacteria thoroughly. In the last step, the Mn concentration in the solution was measured with atomic absorption spectroscopy (AAS). The instrument’s specifications are described in the “Instrumentation” section.

**SEM analysis.** Bacteria were incubated in normal bacteria cultivation medium or in presence of NGA-Mn. Bacteria were incubated for 18 h in 96-well microtiter plates in the same way as described above (standard dilution method). Sub-inhibitory concentration of the NGA-Mn was used in order to observe bacteria before their death, since at higher concentration, no bacteria could be observed, apart from bacterial debris. In the following, bacteria were washed from broth residues by centrifugation, redispersion in PBS, repeated centrifugation and finally were redispersed in water. The bacteria were then fixed on the titanium substrate using a flame, in order to be characterized by scanning electron microscopy. All the samples prepared by this approach were observed by scanning electron microscope (Hitachi SU6600) with acceleration voltage 1.5 kV.

**Construction of fluorescent bacteria.** The Cy5.5 fluorophore was conjugated with a D-amino acid (2,3-diaminopropionic acid), enabling its incorporation into the cell walls of bacteria^4^. Briefly, 200 μL of Cy5.5 in DMSO at a concentration of 5 mg/mL, 60 μL of (S)-3-amino-2-(tert-butyloxycarbonylamino) propionic acid in PBS (pH=8.3, 5 mg mL^-1^), and 240 μL of deionized (DI) water were combined and allowed to react overnight at 4 °C. Following this, the pH of the reaction mixture was adjusted to 2.5 to eliminate the tert-butyloxycarbonyl group. Subsequently, 100 μL of the Cy5.5 reaction mixture was introduced to 1 mL of fresh tryptic soy broth (TSB) medium, which contained 100 μL of mid-exponential growth phase *S. aureus*. The solution underwent incubation at 37 °C under orbital shaking (200 rpm) for 2 h. The resultant labeled *S. aureus* cells were harvested through centrifugation at 8000 g for 5 min, washed three times with saline, and then resuspended in saline to attain a concentration of approximately 10^9^ CFU mL^-1^. The resulting Cy5.5-labeled *S. aureus* cells were utilized for skin wound infection studies.

**Laser scanning confocal microscopy.** *E. coli* cultures were grown in Mueller-Hinton (MH) broth to mid-log phase at 37 °C and incubated with 30 mg L^-1^ NGA-Mn for 24 hours. The bacteria were washed three times with PBS by centrifugation (5000 rcf, 5 min) and suspended in fresh PBS. The bacterial cells were then stained with propidium iodide and SYTO 9 for 15 min and concentrated seven times by centrifugation. Control cultures were incubated in the same manner without adding NGA-Mn and without subsequent concentration step. In the case of positive control, bacteria were treated with 70% isopropanol for 20 minutes. Subsequently, the samples were analysed using an LSM980 laser scanning confocal microscope (Carl Zeiss AG, Germany) equipped with a 63x oil immersion objective.

**Computational details.** Density functional theory (DFT) calculations including geometry optimizations, frequency analysis and binding energy evaluation were performed in Gaussian16^5^ with the B3LYP^6^ functional and D3 empirical dispersion^7^. The main group elements (C, O, N, H) were described with the def2-SVP^8,9^ basis set, while the cations were treated with the LANL2DZ^10^ effective core potential. All calculations included an implicit water solvation model based on the density (SMD)^11^ water model. The models were chosen based on a previous study by Panacek *et al.*^2^

Density functional theory (DFT) calculations were performed with NGA models corresponding to the XPS experimental data in terms of both the composition and the derived spatial proximity of carboxyl groups and nitrogen.

The strength of the interaction of the metal ions to NGA was assessed by the binding energies calculated as $E_{bind}=(E_{NGA+Me}+E_{H_{2}O})-(E_{Me}+E_{NGA})$, where $E_{System}$, $E_{H_{2}O}$, $E_{Me}$, $E_{NGA}$ denote the total energy of the whole studied system, a water molecule, the cation in the form of [Me(H_2_O)_6_]^X+^ (Me/X = Mn^2+^) and the nitrogen-doped graphene-acid model, respectively.

**Molecular dynamics simulations.** All molecular dynamics (MD) simulations were performed by GROMACS 2018^12^ software package using MARTINI 3^13^ force field. Prior to the production run, systems were equilibrated with 2 fs (10 ns long) and 10 fs (10 ns long) time steps. The final production run was performed in the NpT ensemble with 20 fs time step and time scales of 6.37 μs in case of NGA-Mn and 6.0 μs for NGA. The temperature was kept constant at 298 K using the v-rescale thermostat.^14^ The pressure was treated with semiisotropic coupling scheme at 1 atm with Parrinello-Rahman scheme^15^ with coupling constant of 12 ps. Long-range electrostatics were calculated by Particle-mesh Ewald summation^16^ at distance of 1.1 nm, whereas van der Waals interactions were treated with cut-off scheme up to 1.1 nm.

Parameters for NGA-Mn were derived according to the general coarse-grained (CG) MARTINI parametrization workflow. Here, only heavy atoms (omitting hydrogen atoms) were considered for bead representations. The reference data for CG NGA-Mn parameters were taken from our in house all atom (AA) simulations of nitrogen-doped graphene acid. The parametrization workflow comprised of i/ remapping of AA structures into CG bead representation based on the “chemical nature” of included atoms ii/ generating distributions of bonded parameters (bond lengths, angles) from AA simulation corresponding to CG mapping iii/ systematic refinement of CG parameters to match AA distributions. Initial structures for NGA-Mn were generated using online carbon dot builder^17^ and by suitably modifying the protocol for CG output. The composition of NGA-Mn (ratios of C, N and O) was recalculated to reflect the XPS characteristics. NGA-Mn was modelled as single sheet (with dimensions of 15 x 5 nm) containing nitrogen-based beads (represented by TN3a bead type), carbon-based beads (represented by TC5 bead type) and oxygen-based beads (represented as SQ5n (for charged form of carboxylic acid (-1)) and SP2 (for neutral form carboxylic acid (0)). Vacancies (“holes”) on NGA-Mn were presented by TN3a. Each “hole” bead was surrounded by three nitrogen-based beads, which were bound to two negatively charged (type SQ5n) beads. The “hole” bead itself was bound to single Mn^2+^ ion bead (represented as TD bead type, charge +2) compensation the negative charge of carboxylic acids. Neutral forms of carboxylic acids were bond to carbon-based beads (see Scheme S1).


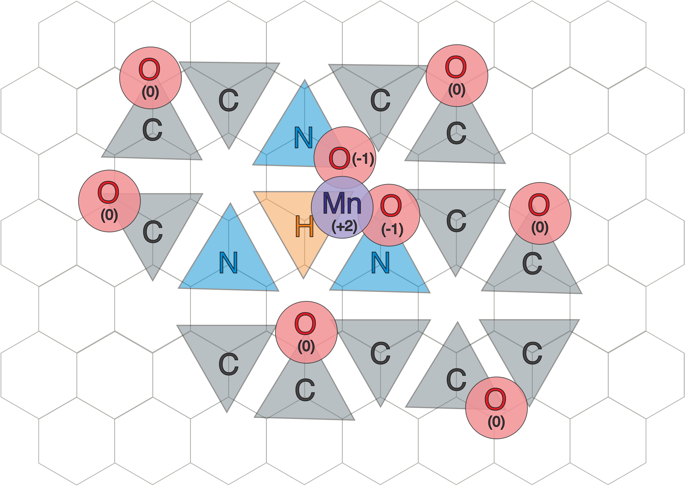


**Scheme S1.** Schematic representation of remapping approach for NGA-Mn. 1^st^ layer is composed of carbon bead, nitrogen-doped beads and “hole” beads. 2^nd^ layer is composed of beads representing carboxylic acids (both charge and neutral states) and 3^rd^ layer comprises of bound Mn^2+^ ions.

Bonded parameters used for NGA-Mn are listed in the table below. As for the angle parameters, for in-plane beads (carbon, nitrogen, and “hole” beads) the angles were set at 120° with force constant of 700 kJ ⋅ mol^-1^ ⋅ rad^-2^, whereas for out-of-plane beads (carboxylic acids connected either to carbon or nitrogen beads) at 90° with force constant of 500 kJ ⋅ mol^-1^ ⋅ rad^-2^. In terms on NGA, the material was modelled without Mn^2+^ ion beads depending upon the material surface.

List of bond length parameters used for NGA-Mn parametrization. Symbols in brackets correspond to bead types for individual beads.

| **Interaction** | **Bond length [nm]** | **Force constant [kJ ⋅ mol^-1^ ⋅ nm^-2^]** |
| --- | --- | --- |
| C (TC5) – N (TN3a) | 0.28 | 7000 |
| C (TC5) – C (TC5) | 0.28 | 7000 |
| N (TN3a) – N (TN3a) | 0.29 | 7000 |
| C (TC5) – O (SP2 (0)) | 0.28 | 5000 |
| N (TN3a) – O (SQ5n (-1)) | 0.28 | 5000 |
| N (“hole”, TN3a) – Mn (TD (+2)) | 0.48 | 8000 |
| Mn (TD (+2)) – O (SQ5n (-1)) | 0.26 | 2000 |

As for the model of bacterial (*Escherichia coli*) membrane, input parameters were taken from previous work of Pluhackova et al.^18^ The composition of membrane model corresponds to inner membrane of *E. coli* at ¾ of the log growth phase, as we focused on non-specific interactions of NGA and NGA-Mn models with the bacterial phospholipids, which is almost identical in the inner and outer phospholipid membrane. It consists of 14 lipid species, out of which 73.6% are phosphatidylethanolamines, 21.8 % are phosphatidylglycerols and 4.6 % are cardiolipins. The great diversity of this membrane model originates from the composition of its acyl chains containing different combinations of saturate, unsaturated and cyclopropanated chains. The membrane composition used in this work reflects the original composition used by Pluhackova et al^18^, but the membrane was enlarged (multiplied by 4) to final *xy-*plane dimensions of 22.5 x 22.5 nm. The initial structures for MD simulations were constructed by placing NGA and NGA-Mn materials above *E. coli* membrane model as represented at Fig. S7. Systems were subsequently solvated and NA^+^ ions were added to retain the electroneutrality of simulation boxes.

In addition, 1 µs of unbiased MD simulations of small NGA and NGA-Mn models (material flake sizes of 2x2 nm) on bacterial membrane model composed of lipopolysaccharides (LPS) [[Vaiwala, R.; Ayappa, K. G. *J. Chem. Theory Comput.* **2024**, *20*, 1704](https://doi.org/10.1021/acs.jctc.3c00471)] were performed. Flat bottom potential was applied to restrict the interaction to outer layer of bacterial membrane containing LPSs as described in [[Biriukov, D.; Javanainen, M. *J. Chem. Theory Comput.* **2023**, *19*, 6332](https://doi.org/10.1021/acs.jctc.3c00614)].

All analysis was performed by tools implemented in GROMACS software package. Density profiles were calculated using *gmx density* tool along the membrane normal. Minimal distance between NGA-Mn and the model of bacterial membrane was calculated by *gmx mindist* tool. 2D density maps of individual lipid species and materials were calculated using *gmx densmap* tool and the plots were generated from the last 3.0 μs of final production runs. All figures were prepared and rendered using PyMOL visualization software.^19^

In order to evaluate the affinity of NGA and NGA-Mn we performed potential of mean force (PMF) calculation. We prepared small NGA and NGA-Mn models (2 x 2 nm) and a small membrane models (with the same *E. coli* lipid ratio corresponding to 64 lipids per leaflet). With the same simulation setup as with larger models, we performed 1 µs of unbiased simulation and extracted snapshots separated by 0.1 nm in the position of NGA(-Mn) flake. Further, we performed umbrella sampling simulations for 500 ns per window, calculated PMF for each 100 ns to evaluate its convergence. We set the first 200 ns as an equilibration phase and calculated final PMF starting at 200 ns of umbrella simulations.

**Instrumentation.** The materials (NG, NGA and NGA-Mn) were characterized by transmission electron microscopy (TEM) using a JEM 2010 TEM instrument (Jeol, Japan).

The concentration of Mn in NGA-Mn dispersion was measured in an atomic absorption spectroscopy (AAS), on a ContrAA 600 with graphite furnace (Analytik Jena AG, Germany) equipped with a high-resolution Echelle double monochromator (spectral band width, 2 pm at 200 nm) and a xenon lamp as a continuum radiation source. In order to confirm the measurement, the concentration was further measured using 7500ce inductively coupled plasma mass spectrometry (ICP-MS) instrument (Agilent).1 mg (weighted on a six decimal digit balance) of the material was added to a 5% nitric acid solution to form a final solution of 50 ml. The solution thus prepared was sonicated for 3 h in order to unbind all Mn ions from the graphene surface. After sonication, the solution was filtered using a 100 nm filter to remove all graphene material from the solution. Subsequently, the filtrate was collected and measured by ICP-MS and AAS to determine the amount of Mn that was bound to NGA-Mn. In order to determine the leaching of Mn ions from NGA-Mn, 10 mg of the composite was dispersed in 4 mL of distilled water and 4 mL of culture medium, followed by non-shaking for 24 hours and shaking for 24 and 72 hours. Then, it was filtered using a syringe filter (0.1 μm pore size) to obtain the possibly released Mn ions. After filtration, in order to dissolve the possibly present clusters, 2 mL of filtrate were put to 50 mL of 2% nitric acid and sonicated for 30 min. The quantity of released manganese in the filtrate was determined with AAS.

FTIR spectra were recorded on an iS5 FTIR spectrometer (Thermo Nicolet) using the Smart Orbit ZnSe ATR accessory. Briefly, a droplet of an ethanol dispersion of the relevant material was placed on the ZnSe crystal and dried. The spectra were then acquired by summing 52 scans while using a nitrogen gas flow through the ATR accessory. ATR and baseline correction were applied to the collected spectra.

The sample morphology was examined through scanning electron microscopy (SEM) JEOL 7900F microscope (JEOL, Japan), with accelerating voltage of 5 kV. Dynamic light scattering was also performed with a Zetasizer Nano ZS instrument (Malvern), providing a mean hydrodynamic diameter for NGA-Mn of ca. 150 nm in distilled water and ca. 160 nm in the presence of proteins, salts and aminoacids (EMEM culture medium containing serum).

Survey and high-resolution X-ray photoelectron spectroscopy (HR-XPS) was carried out with a PHI VersaProbe II (Physical Electronics, Japan) spectrometer using an Al K_α_ source (15 kV, 50 W, spot size is 100 µm in diameter). The survey spectrum was acquired using pass energy 187.85 eV, step size 0.8 eV, the HR-XPS spectra were acquired using pass energy 23.5 eV, step size 0.2 eV. Elemental analysis was obtained from high resolution scans of the respective elements. The obtained data were evaluated and deconvoluted with the MultiPak (Ulvac - PHI, Inc.) software package. The spectral analysis process involved Shirley background subtraction and peak deconvolution using mixed Gaussian–Lorentzian functions. All binding energies are referenced with respect to C-C bond at 284.8 eV.

HR-TEM images were obtained using a HR-TEM TITAN 60-300 microscope with an X-FEG type emission gun, operating at 300 kV. Scanning transmission electron microscopy high-angle annular dark-field imaging (STEM-HAADF) analysis for EDS (energy-dispersive X-ray spectroscopy) elemental mapping on the products was performed with a FEI Titan HR-TEM microscope operating at 80 kV. For this analysis, a droplet of an aqueous dispersion of the material under study with a concentration of ~0.1 mg mL^–1^ was deposited on a carbon-coated copper grid and dried at room temperature for 24 hours.

X-ray powder diffraction (XRD) was measured employing Aeris diffractometer (Malvern PANalytical) in parafocusing Bragg-Brentano geometry using iron filtered Co *K_α_* radiation source. The diffractometer is equipped with fixed divergence and diffracted beam antiscatter slits and PixCell detector. The powder sample (NGA and NGA-Mn) were prepared on zero-background Si slide and measured in 2*θ* range from 5 to 105° with total counting time 64 min. Commercially available standard SRM640 (Si) was used for evaluation of line positions. The data were processed using HighScore Plus software in conjunction with PDF-4+database.

EPR spectra were collected on X-band (∼9.14–9.17 GHz) spectrometer JEOL JES-X-320 equipped by variable He temperature set-up ES-CT470 apparatus. Experimental temperature was set to 78 K. The quality factor (Q) was kept above 6500 for all measurements to make the spectra comparable. As a sample holder were used high purity quartz tubes (Suprasil, Wilmad, ≤0.5 OD) and the accuracy of the g-values was determined by comparison with a Mn^2+/^MgO standard (JEOL standard). The microwave power was set to 1.0 mW to avoid any power saturation effects. A modulation width of 1-0.25 mT and a modulation frequency of 100 kHz were used. All EPR spectra were collected with a time constant of 30 ms and a sweep time of 2 min with two accumulations to improve the signal-to-noise ratio. For all experiments the EPR tubes were loaded with 100 µl of solution containing the NGA or NGA-Mn (0.5 mg mL^-1^) and with Mn^2+^ solution (10 ppm and 10 ppb). For the experiment to determine the interaction of NGA-Mn with *Escherichia coli* (without and with medium-EPR inactive), EPR tubes were filled with 100 µl of a solution containing NGA-Mn (0.5 mg ml^-1^) with bacterial solution, following the protocol described in the ''antibacterial assay'' section. Spectra were then measured at time 0 and after 18 hr.

**X-ray Absorption Spectroscopy.** X-ray absorption fine structure (XAFS) spectra of Mn K-edge were carried out at BL13SSW beamline station and supported by the user experiment assist system in Shanghai Synchrotron Radiation Facility (SSRF). All samples were measured under transmission mode using Si (311) double-crystal monochromator. The acquired EXAFS data were analyzed according to the standard procedures using the ATHENA module implemented in the IFEFFIT software packages (J Synchrotron Radiat. 12 (2005) 537-541). Investigation of the interaction of the antibacterial agent NGA-Mn with *E. coli* bacteria: For this purpose, a solution of *Escherichia coli* (bacteria were placed in ultrapure water at a concentration of 5x10^5^ cfu/mL, without the presence of any medium) was incubated with NGA‑Mn (sub-inhibitory concentration) according to the protocol described above (Antibacterial assay). Finally, the solution containing the NGA-Mn with bacteria was dried in lyophilizer and submitted for measurement.

**Supplementary Figures and Tables.**


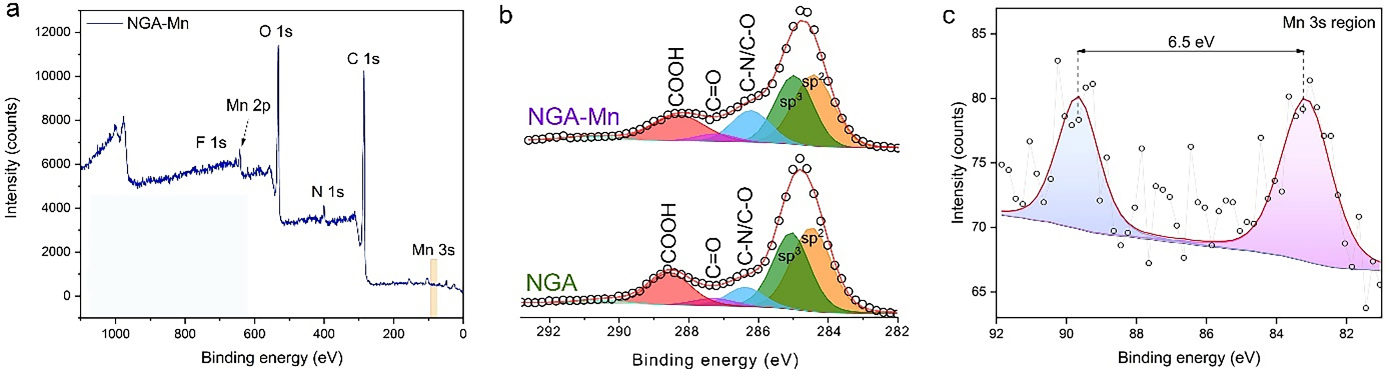


**Figure S1.** (a) Survey spectra of NGA-Mn and (b) deconvoluted HR-XPS of the C 1s region, (c) HR-XPS of Mn 3s region. Owing to the effects of highly electronegative functional groups^20^ in the NGA-Mn, the energy splitting is slightly higher than those in a regular MnO^21^. Further comments on the XPS results: The deconvolution of the C 1s region in NGA revealed extensive bonding of carbon to oxygen and nitrogen corresponding to -COOH (16.5 at. %), C=O (3.8 at. %) and C-O/C-N (8.9 at. %) groups (Fig. S1b and Supplementary Table S1). The immobilization of manganese cations on the NGA (4.5 wt. % according to inductively coupled plasma mass spectrometry) surface was performed by treating the NGA with manganese nitrate. XPS N 1s of the starting material NG, and after its treatment with nitric acid producing the NGA is available on page 12 in the supporting information of the published work (Panáček, D. et al. Small 2022, 18, 2201003)^2^.

**Table S1.** Deconvoluted carbon components for the HR-XPS C 1s region of NGA and NGA‑Mn materials.

|  | Binding states | | | | | |
| --- | --- | --- | --- | --- | --- | --- |
|  | sp^2^ | sp^3^ | C-N/C-O | C=O | COOH | C-F and satellite |
| NGA | 36.2 | 32.4 | 8.9 | 3.8 | 16.5 | 2.15 |
| NGA-Mn | 33.7 | 29.3 | 14.1 | 3.8 | 16.7 | 2.4 |


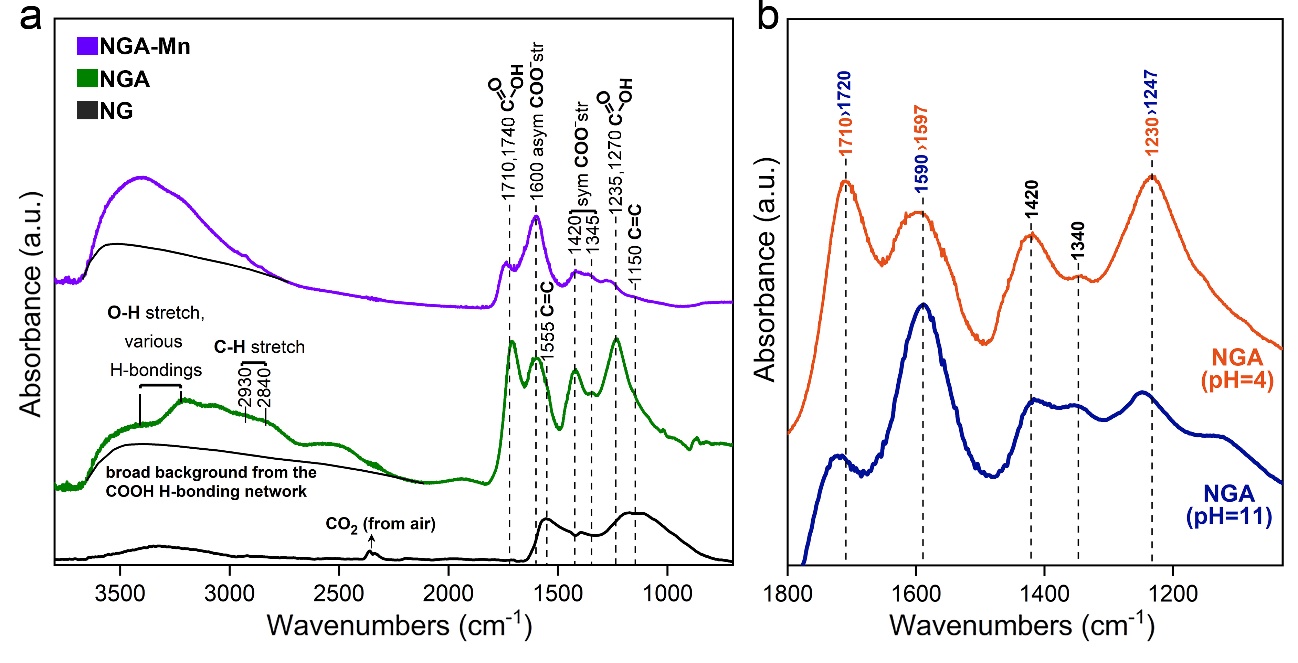


**Figure S2.** **(a)** Comparison of the whole FTIR spectra of NG, NGA, and NGA-Mn materials. **(b)** Comparison of fingerprint regions of NGA FTIR spectra measured from dispersions with pH of 4 and 11 adjusted by addition of NaOH. The position was shifted to 1720 cm^-1^. This may be caused by the restriction of C=O vibration by the sodium cations anchored to the carboxyl groups. *Further comments on the FTIR results:* **panel (a)** FTIR analysis revealed the presence of skeletal vibrations of sp^2^-hybridized carbon lattice in NG (at 1555 cm^−1^ and in the area between 1000 and 1250 cm^-1^,^2,22,23^ along with vibrations related to nitrogen-containing aromatic areas. The latter appear clearly in the NG product (see the designated band at 1395 cm^−1^ in Fig. 1d in the main manuscript). In NGA, the broad band between 1000 cm^−1^ and 1250 cm^−1^ changed its pattern in comparison to NG, comprising now also the C‑O stretching modes from the carboxylic groups.^24^ The broad feature ranging from 2300  cm^−1^ to 3500 cm^−1^ reflects the network of O−H stretching vibrations in various hydrogen bonding configurations. **Panel (b)** Ionization of carboxyl groups of NGA by adjusting pH of the dispersion to 11 using NaOH caused substantial suppression of the 1710 cm^-1^ and 1235 cm^-1^ bands associated with the carboxylic[C(=O)OH] and C-OH stretching vibrations of the carboxylic groups, and strengthened the asymmetric carboxylate anion stretch at 1590 cm^-1^, confirming that these vibrations indeed correspond to carboxyl groups.


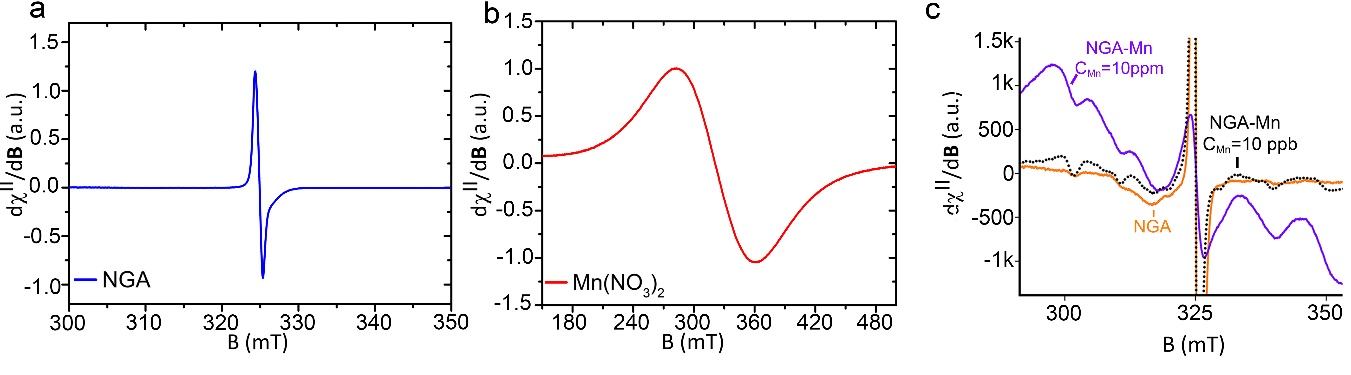


**Figure S3.** CW X-band EPR spectra of (a) NGA material, (b) Mn(NO_3_)_2_ salt. (c) Comparison of the EPR spectra of NGA, NGA loaded with 10 ppm of Mn^2+^ and NGA loaded with 10 ppb of Mn^2+^ (magnification of Fig. 1d from main text of MS). *Further comments:* In the NGA-Mn sample with a low concentration of Mn^2+^ (10 ppb), the decrease in NGA’s signal intensity is characterized by the lack of changes in the resonance linewidth signal, indicating that manganese cations interact with spin containing sites in the NGA backbone via strong antiferromagnetic coupling, giving NGA‑Mn^2+^ system (S_eff_ = 2) with transitions that cannot be resolved at X-band frequency.


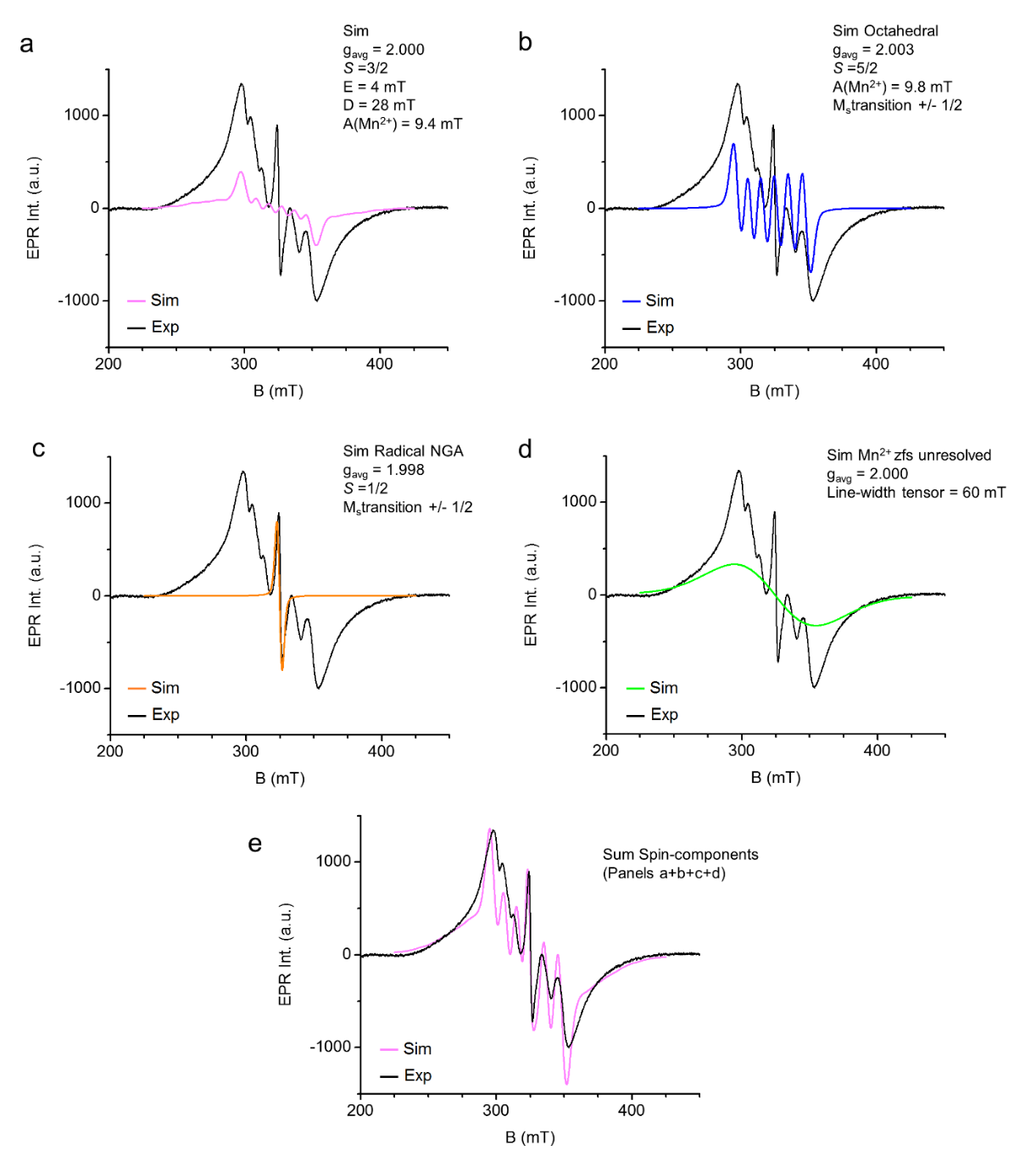


**Figure S4.** EPR simulations (colour lines, panels **a** to **e**) of the various spin-components observed in the CW X-band EPR spectrum, 10 ppm Mn^2+^ and NGA (experimental, black line, recorded at *T* = 80 K in Fig. S3c). (a): EPR envelope simulation of the *M*s ±3/2 Kramer doublets (pink line), that include axial (D) and rhombic (E) zero-field splitting energy, together with the nuclear hyperfine term (*A*) of the ^55^Mn^2+^ cation. Calculated values for D, E, A are given inside the panel’s legend; (b): EPR envelope simulation of the *M*s ±1/2 transition (blue line) associated to the high spin ^55^Mn^2+^ cation including nuclear hyperfine term; (c): EPR envelope simulation of the *M*s ±1/2 transition (orange line) associated to the radical species (*S* = ½) found in neat NGA; (d): EPR envelope simulation illustrating unresolved *M*s ±3/2, ±5/2 transitions originating from ^55^Mn^2+^ cation in high spin state (green line); (e) shows the overall EPR envelope simulation that results from the sum of all spin components given in panels a to d. Respective signal intensity weights used in the simulations were 18% (as given in panel (a)), 31.4% (as given in panel (b)), 35.9% (as given in panel (c)) and 14.7% (as given in panel (d)). Simulations of the powder EPR spectra were carried out with the WinEPR SimFonia software (V.1.25, *EPR Division*, Bruker Instruments, Inc., Billerica, USA) using second-order perturbation theory and spherical integration (grid) of 200 (theta), 200 (phi), with Lorentzian-Gaussian line ratio of 0.63. *Further comments:* In this context, antiferromagnetic spin exchange coupling (*S*_eff_=2) between Mn^2+^(*S*=5/2) cations with the radical sites (*S*=1/2) belonging to NGA becomes so effective that cannot be resolved at X-band frequency. High-spin Mn^2+^ exists in an ^6^S electronic state and when bound to an organic scaffold, such as NGA, the 6-fold degeneracy is expected to be lifted, generating zero-field splitting (*zfs*) terms among the three Kramers doublets (*M*s ±1/2, ±3/2, ±5/2), which can give large signal complexity to the overall EPR spectrum, with 30 possible allowed transitions (Δ*M*s ±1, Δ*M*_I_ ±0). Even along the *M*s ±1/2 transition, expected to emerge at g ~ 2.00, the resonance line can be severely broadened by overlapped *zfs*-components (Fig. S4a and Fig. S4d). Nevertheless, the effective coordination of Mn^2+^ to NGA is clearly revealed around g= 2.00 by the appearance of characteristic set of resonant lines (sextet) arising from the nuclear hyperfine coupling (^55^Mn, *I*=5/2, 100%) originating from the lowest Kramer doublet (+1/2→−1/2) of mononuclear Mn^2+^. The estimated ^55^Mn nuclear hyperfine coupling from EPR simulation (A_iso_ = 9.8 mT, Fig. S4b) is in harmony with those observed in hexacoordinate Mn^2+^ enzymes. containing a set of N,O donors in distorted octahedral geometries such as oxalate oxidase (~9 mT)^25^, concanavalin A (9.3 mT)^26^, manganese catechol dioxygenase (9.5 mT)^27^, manganese lipoxygenase (9.5 mT)^28^, and oxalate decarboxylase (9.5 mT).^29^

**Table S2.** Second-order perturbative estimates of the donor-acceptor interactions and the natural electron configuration of NGA with the Mn^2+^ cation. LP/LP* stands for a valence lone pair to the Lewis/non-Lewis natural bond orbitals.

| **Donor** | **Acceptor** | **E(2) (kcal mol^-1^)** |
| --- | --- | --- |
| **NGA** | **ION** |  |
| (LP) N10 | (LP^*^) Mn56 | 12.08 |
| (LP) N35 | (LP^*^) Mn56 | 11.65 |
| (LP) N3 | (LP^*^) Mn56 | 7.42 |
| (LP) N35 | (LP^*^) Mn56 | 6.53 |
| (LP) N3 | (LP^*^) Mn56 | 5.75 |
| (LP) N10 | (LP^*^) Mn56 | 5.30 |
| (LP) N10 | (LP^*^) Mn56 | 4.88 |
| Mn: [core] 4s^0.15^3d^5.13^4p^0.35^4d^0.01^5p^0.02^ | | |

**Table S3.** The six highest values of Wiberg bond indices (WBI) estimated using the natural atomic orbital basis for the ion–NGA atom pairs. The total Wiberg bond index (TWBI) is the sum of all Wiberg indices for the probed ion.

| **Mn^2+^** | |
| --- | --- |
| **Atom** | **WBI** |
| O60 | 0.0630 |
| O57 | 0.0617 |
| O64 | 0.0577 |
| N10 | 0.0555 |
| N35 | 0.0547 |
| N3 | 0.0364 |
| **Atom** | **TWBI** |
| Mn56 | 0.3983 |

**Table S4.** X-ray absorption spectroscopy results of NGA-Mn confirm the presence of only single Mn^2+^ atoms on NGA surface and their coordination with nitrogen and oxygen moieties.

| **Sample** | **Shell** | ***N*^[a]^** | ***R*^[b]^ (Å)** | ***σ*^2[c]^×10^3^ (Å^2^)** | **Δ*E*_0_^[d]^ (eV)** | ***R* factor (%) ^[e]^** |
| --- | --- | --- | --- | --- | --- | --- |
| NGA-Mn | Mn-N/O | 5.2±0.5 | 2.0-2.2 | 0.001 | -6.1±2.5 | 0.07 |

[a] the coordination number for the absorber-backscattered pair. [b] the average absorber-backscattered distance. [c] the Debye-Waller factor. [d] the inner potential correction. [e] goodness of fit. *s*_0_^2^ was fixed to 0.92 as determined from MnO fitting.


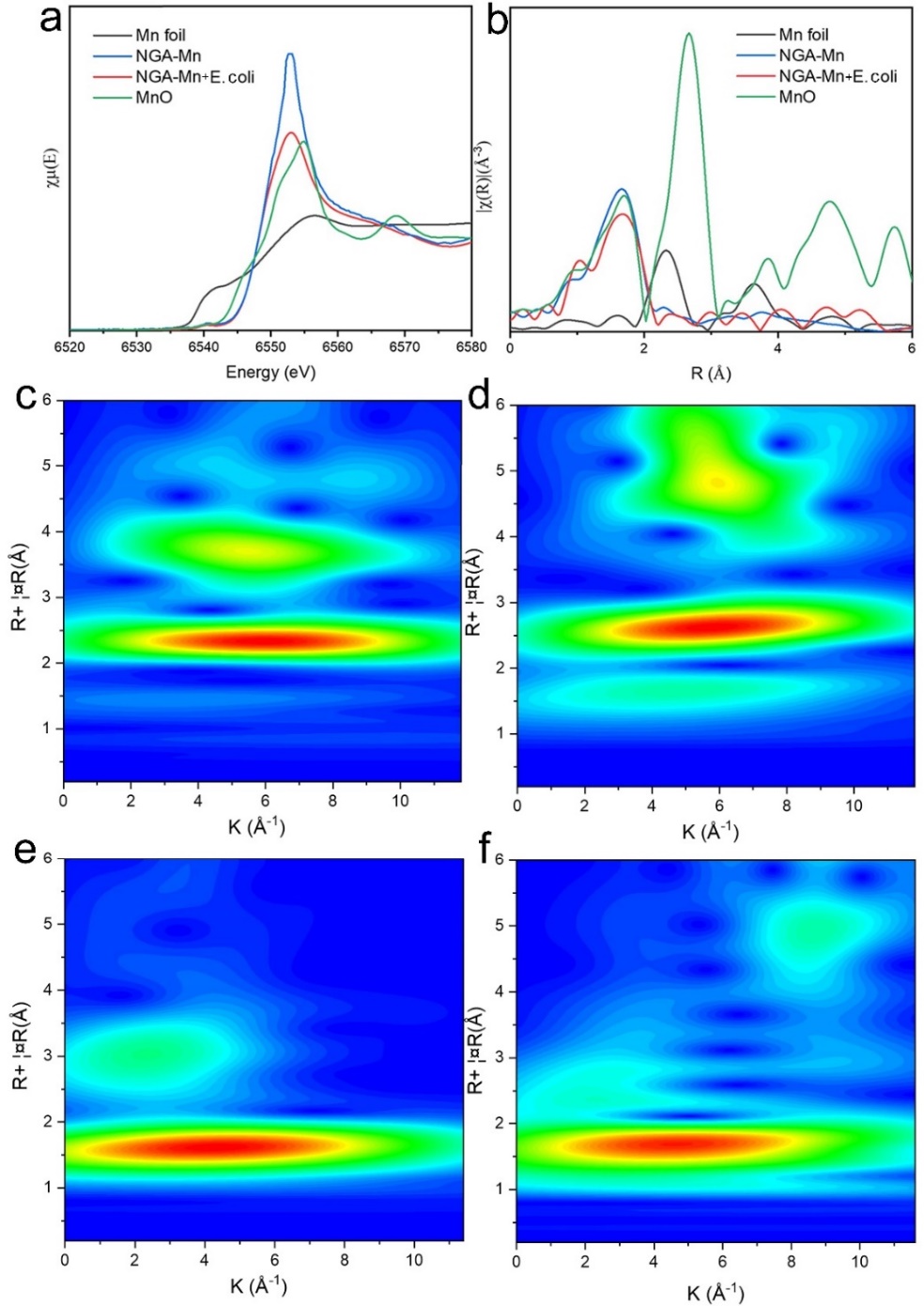


**Figure S5.** (a) XANES, and (b) *k*^2^ weighted FT EXAFS in R space for Mn foil, MnO, NGA‑Mn pure and in presence of *Escherichia coli* (NGA Mn+E. coli). Wavelet transformation for the *k*^2^-weighted EXAFS signal of the (c) Mn metallic foil, (d) MnO, (e) NGA‑Mn, and (f) NGA‑Mn+E.Coli. *Further comments:* X-ray absorption fine structure (XAFS) measurements were performed to study the coordination of Mn species immobilized on NGA. As shown in Fig. S5a, Mn K-edge XANES spectra show that the absorption edge of NGA-Mn is higher than that of Mn foil, indicating the oxidation state of Mn species. The FT-EXAFS spectra reveal the absence of significant scattering peaks beyond the first coordination shell of the NGA-Mn (Fig. S5b), verifying the absence of the Mn–Mn bonds. EXAFS fitting cannot explicitly distinguish N and O due to the similarity of their scattering paths.^30^ However, it could be concluded from the combination of XPS, EPR, and DFT results that the Mn atom in NGA-Mn is coordinated to both N and O. Furthermore, one intensity maximum at ca. 6 Å^-1^ is observed in the WT-EXAFS of Mn foil (Fig. S5c) and at radial distance above 2 Å, corresponding to Mn-Mn bond. Nevertheless, the main intensity maximum for NGA-Mn is about 4.2 Å^-1^ (Fig. S5e) at radial distance bellow 2 Å, assigned to Mn–N/O, confirming the atomic dispersion of Mn atoms. The WT-EXAFS of the NGA‑Mn interacting with E. coli (Fig. S5f) demonstrate the preservation of the initial coordination environment, but with particularly affected scattering profile at higher radial distances beyond the first coordination sphere, reflecting the complex coordination environment imposed by the surrounding biomolecules. The first coordination sphere is practically identical, since the light atoms of the biomolecules do not differentiate in the XANES/EXAFS first coordination sphere from the light atoms of NGA and water, where Mn is initially coordinating with.


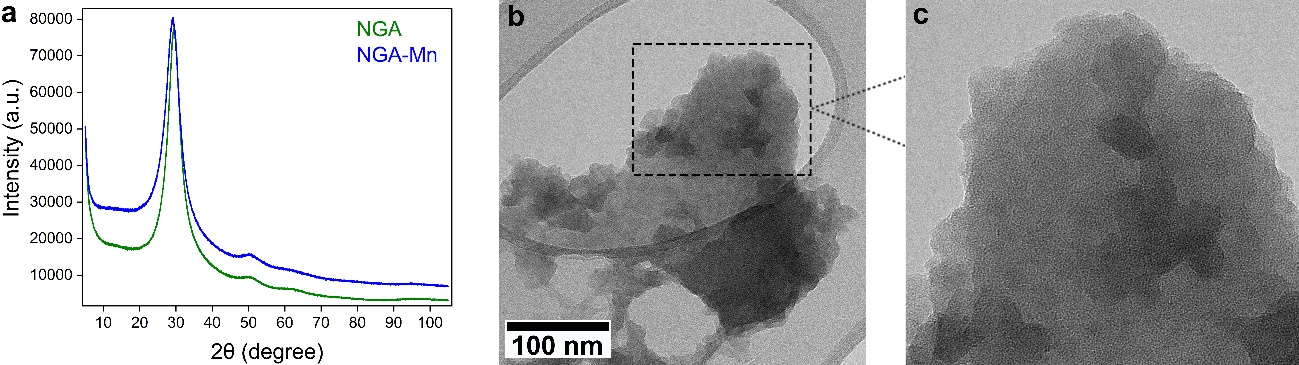


**Figure S6.** (a) XRD measurement of NGA and NGA-Mn. The broad reflection at 29.5 two theta degrees corresponds to 0.35 nm d-spacing arising from disordered graphene regions with parallel stacking. Transmission electron microscopy of (b) NGA-Mn material and (c) detailed zoom of NGA-Mn showing a few layered structures of graphene sheet.

**Table S5.** MIC values of NGA-Mn, NGA, Mn(NO_3_)_2_ salt, and NGA-Mn in presence of albumin against drug-susceptible and multidrug-resistant bacteria.

| Bacteria strain | Type of resistance | NGA-Mn^a^ | | NGA | Mn(NO_3_)_2_ | NGA-Mn  +albumin |
| --- | --- | --- | --- | --- | --- | --- |
|  |  | MIC  (mg L^−1^) | MBC  (mg L^−1^) | MIC  (mg L^−1^) | MIC  (mg L^−1^) | MIC  (mg L^−1^) |
| *Escherichia coli*  CCM 3954 |  | 90 (4) | 90 (4) | >1500 | >1500 | 180 (8) |
| *Staphylococcus epidermidis* CCM 7221 |  | 90 (4) | 180 (8) | >1500 | >1500 |  |
| *Pseudomonas aeruginosa* CCM 3955 |  | 90 (4) | 90 (4) | >1500 | >1500 |  |
| *Staphylococcus aureus* CCM4223 |  | 355 (16) | 710 (32) | >1500 | >1500 | 710 (32) |
| *Enterococcus faecalis*  CCM 4224 |  | 355 (16) | 355 (16) | >1500 | >1500 |  |
| *Candida albicans*  CCM 8161 |  | 180 (8) | 355 (16) | >1500 | >1500 |  |
| *Staphylococcus aureus* 4591/A | Methicillin-resistant | 355 (16) | 355 (16) | >1500 | >1500 |  |
| *Enterococcus faecium* 419/ANA | vancomycin-resistant | 355 (16) | 355 (16) | >1500 | >1500 |  |
| *Escherichia coli*  CE5556 | multidrug-resistant  (CTX-M-15, gyrA, aminoglycoside resistant) | 180 (8) | 355 (16) | >1500 | >1500 |  |
| *Pseudomonas aeruginosa* 21425/C | multidrug-resistant  (PDC+) | 90 (4) | 180 (8) | >1500 | >1500 |  |
| *Escherichia coli*  NCTC 13846 | extended-spectrum beta-lactamase  (CTX-M-15) | 180 (8) | 355 (16) | >1500 | >1500 |  |
| *Klebsiella pneumoniae* NCTC 13438 | carbapenemase production  (KPC-3 carbapenemase) | 355 (16) | 355 (16) | >1500 | >1500 |  |
| *Klebsiella pneumoniae* NCTC 13443 | (NDM-1)  resistant to penicillin, cephalosporins, and carbapenems | 355 (16) | 355 (16) | >1500 | >1500 |  |
| *Acinetobacter baumannii* NCTC 13301 | (OXA-23, OXA-51-like) | 90 (4) | 180 (8) | >1500 | >1500 |  |
| *Escherichia coli*  NCTC 13353 | colistin resistant (mcr-1) | 180 (8) | 180 (8) | >1500 | >1500 |  |
| *Enterobacter cloacae* NCTC 13406 | extended-spectrum beta-lactamase  (AmpC beta-lactamase) | 90 (4) | 90 (4) | >1500 | >1500 |  |

**^a^** Values refer to the mass of NGA-Mn and values in parentheses refer to the MIC values with respect to the mass of the Mn only, according to its content in the hybrid. The Mn content in NGA-Mn hybrid was 4.5 wt.%.

MIC = Minimum Inhibition Concentration, MBC = Minimum Bactericidal Concentration, CCM = Czech Collection of Microorganisms, NCTC = National Collection of Type Cultures (UK).

Comment. We performed experiments for MIC determination using NGA-Mn, but deliberately changed the Mn content from 4.5wt.% of the original NGA-Mn to 1.4 wt.% and 2.6 wt.%. The results demonstrated a significant increase in MIC from 4 mg_Mn_ L^-1^ (for NGA-Mn with Mn content of 4.5 wt.%) to 16 and 64 mg_Mn_ L^-1^ for NGA Mn with Mn content of 2.6 and 1.4 wt.%, respectively.

**Table S6.** MIC values of the NGA-Mn hybrid compared with state-of-the-art antibacterial agents.

| Antibacterial agent | Incubation time  [h] | MIC^a^  [mg L^–1^] | | | | Cytocompati-bility for  human cells^b^  [mg L^–1^] | Resistance  (cycles) | ref. |
| --- | --- | --- | --- | --- | --- | --- | --- | --- |
|  |  | ***E.coli*** | ***S.aureus*** | ***K. pneumoniae*** | ***A. baumannii*** |  |  |  |
| NGA-Mn | 18 | 90**^c^**  100% | 355**^c^**  100% | 355**^c^**  100% | 90**^c^**  100% | 2200  100% | 30 | **This work** |
| Bi_2_S_3_/Ti_3_C_2_Tx MXene | 0.17 | 200  100% | 200  100% | NA | NA | 200  100% | NA | ^31^ |
| GMO-LL-37 | 18 | 64  ~85% | inactive | NA | NA | 64  80% | NA | ^32^ |
| Guanidine Nanogels | 11 | NA | 120  90% | NA | NA | 120  100% | NA | ^33^ |
| Si sheets | NA | 200  100% | 200  100% | NA | NA | NA | NA | ^34^ |
| CaO_2_ | 18 | 100  100% | NA | NA | NA | NA | NA | ^35^ |
| Pd@Ir | 12 | 25  ~60% | NA | NA | NA | NA | NA | ^36^ |
| Comparisons with molecular antibiotics | | | | | | | | |
| Antibacterial agent | **Incubation time**  **[h]** | **MIC^a^**  **[mg L^–1^]** | | | | **Cytocompati-bility for**  **human cells^b^**  **[mg L^–1^]** | **Resistance**  **(cycles)** | **ref.** |
|  |  | ***E. coli*** | ***S. aureus*** | ***K. pneumoniae*** | ***A. baumannii*** |  |  |  |
| NGA-Mn | 18 | 4**^d^**  100% | 16**^d^**  100% | 16**^d^**  100% | 4**^d^**  100% | 100**^d^**  100% | 30 | **This work** |
| macolacin | 16 | NA | inactive | 1  100% | 1  100% | 2  100 % | NA | ^37^ |
| synthetic molecule  ETX0462 | 36 | 1  90% | inactive | 4  90% | 4  90% | NA | NA | ^38^ |
| arylomycin peptides | NA | 0.13  100% | 0.06  100% | 0.13  100% | 1  100% | 41  100% | 1 | ^39^ |
| teixobactin | 18 | 25 | 0.25 | >40 | NA | 100  100% | 27 | ^40^ |

**^a,b^**The percentiles appearing bellow the MIC values refer to cases where the bacterial growth inhibition was not 100%, or where cytocompatibility did not refer to 100% viability of the cells. “NA” stands for unavailability of data.

**^C^**For the comparisons with antibacterial nanomaterials the MIC values with respect to the full mass of NGA‑Mn was used in order to allow comparisons with these materials

**^d^** MIC values for the NGA‑Mn expressed with respect to the concentration of Mn^2+^ in order to allow comparisons with the molecular antibiotics that do not contain any carrier/matrix.


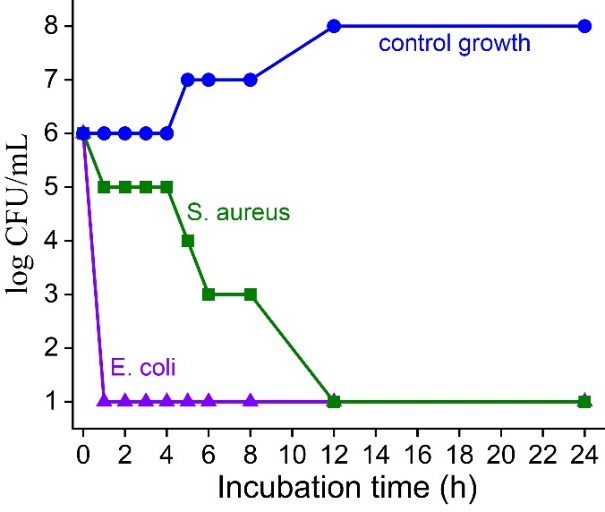


**Figure S7.** Time-dependent antibacterial assessment for NGA-Mn.


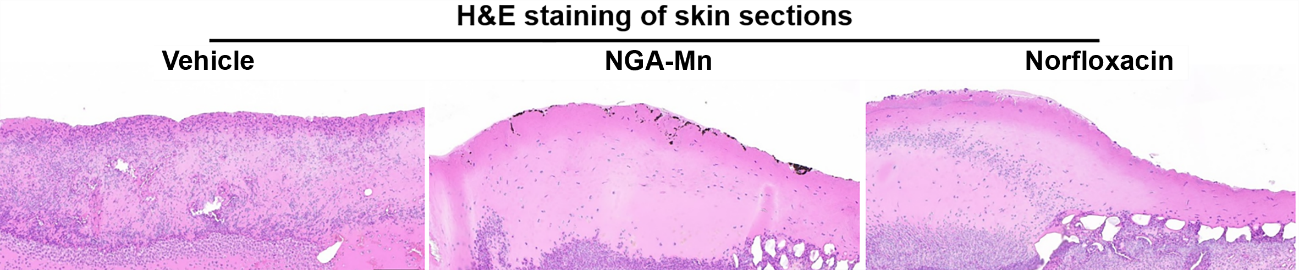


**Figure S8.** Representative images of haematoxylin and eosin (H&E) staining on skin sections. Washing fluids from skin wounds of mice infected with *S. aureus* were collected on the 6^th^ day post-treatment to detect pro-inflammatory cytokines using ELISA (n=3). Additionally, healed skin samples were subjected to H&E staining for histological examination. **p*<0.05, ***p*<0.01, *** *p*<0.001 compared to vehicle control by Student t test.

Comment: In another approach, to simulate *in vivo* conditions, MIC was measured in the presence of 4 wt.% albumin. The MIC concentration increased only by twofold (Supplementary Table S5). This increase, corresponding to only one dilution step, is marginal since it is accepted that even under the most scrupulous conditions, there is an uncertainty of ±1 2-fold concentration increment.^41^ As confirmed by the *in vivo* studies, the MIC in presence of albumin also suggest the practical applicability of NGA-Mn. It is also interesting to note that the MIC value of a common molecular antibiotic (minocycline) increases substantially in the presence of serum by 16-fold.^42^


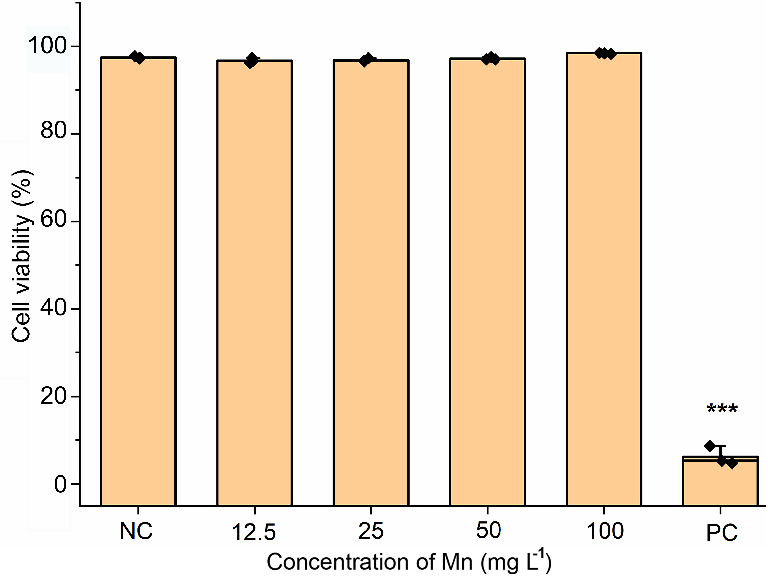


**Figure S9.** Viability of THP-1 treated with various concentrations of NGA-Mn for 24 h. Heat killed cells were used as a positive control (PC). One-way ANOVA with Dunnett’s post hoc test was applied for statistical significance (n = 3).∗∗∗p ≤ 0.001.


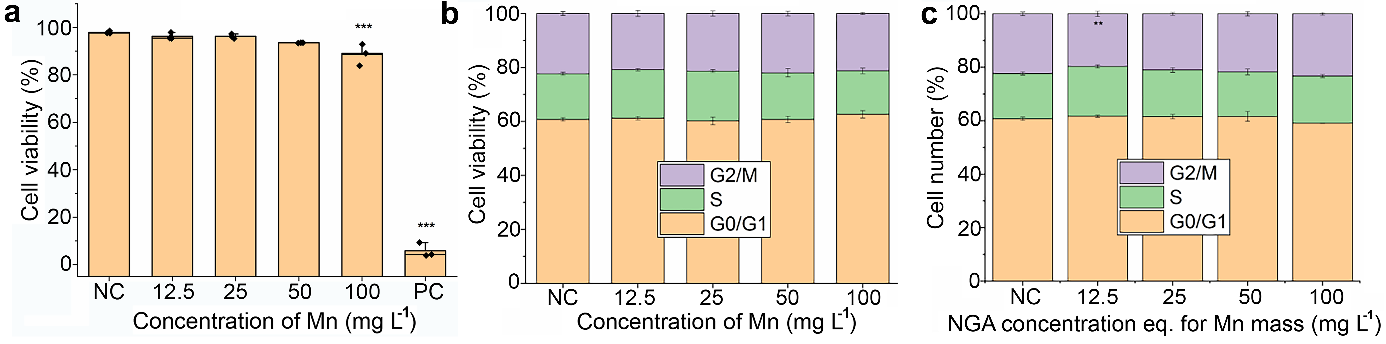


**Figure S****10.** a) Viability of THP-1 cells treated with various concentrations of NGA-Mn for 24 h in culture medium without supplementation of FBS (i.e., proteins). Heat killed cells were used as a positive control (PC). b,c) Cell cycle analysis of THP-1 cells treated with various concentrations of NGA-Mn (b) or NGA alone (c) (equivalent concentration for Mn mass in NGA-Mn considering the Mn content of 4.5 wt.%) for 24 h. One-way ANOVA with Dunnett’s post hoc test was applied for statistical significance (n = 3). ∗p ≤ 0.05, ∗∗p ≤ 0.01, ∗∗∗p ≤ 0.001.


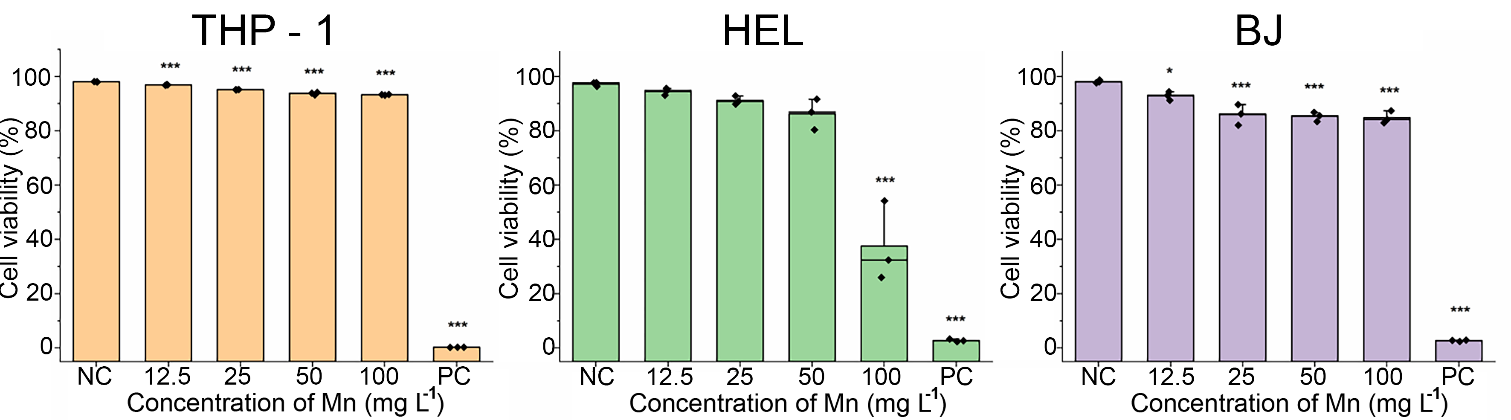


**Figure S11.** Viability of THP-1, HEL and BJ cells treated with various concentrations of NGA-Mn for 72 h. Heat killed cells were used as a positive control (PC). One-way ANOVA with Dunnett’s post hoc test was applied for statistical significance (n = 3). ∗p ≤ 0.05, ∗∗∗p ≤ 0.001. *Further comments on the biological results:* In the HEL cell line, significant cytotoxicity was detected only in sample treated with the highest concentration (100 mg_Mn_ L^‑1^), leading to a viability drop to 40 %. Nevertheless, NGA-Mn exhibited remarkable biocompatible properties even in this cell model, with a non-toxic concentration of 50 mg_Mn_ L^‑1^ considerably surpassing the antibacterial MIC values 4 - 16 mg L^-1^ (Fig. 3a).


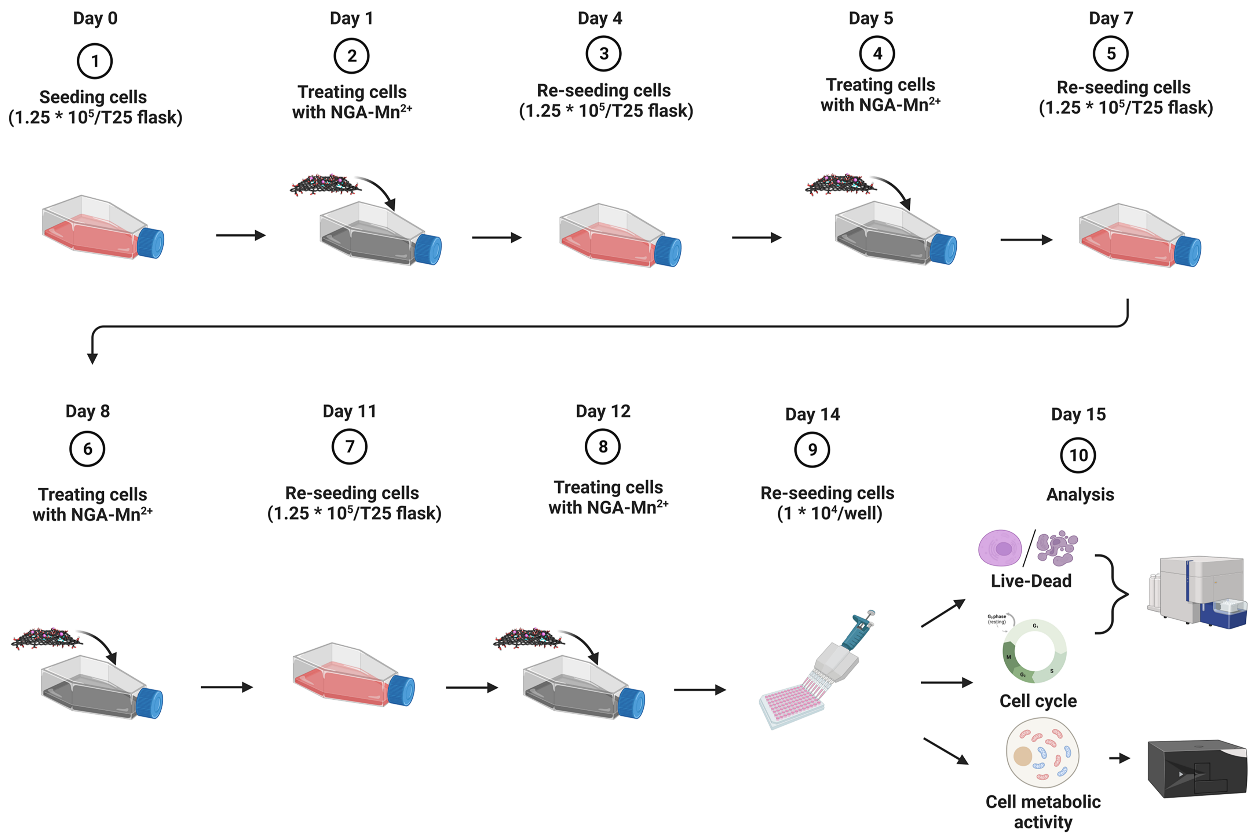


**Figure S12.** The long-term safety assessment for NGA-Mn (cumulative dose of 10 mg L^-1^).


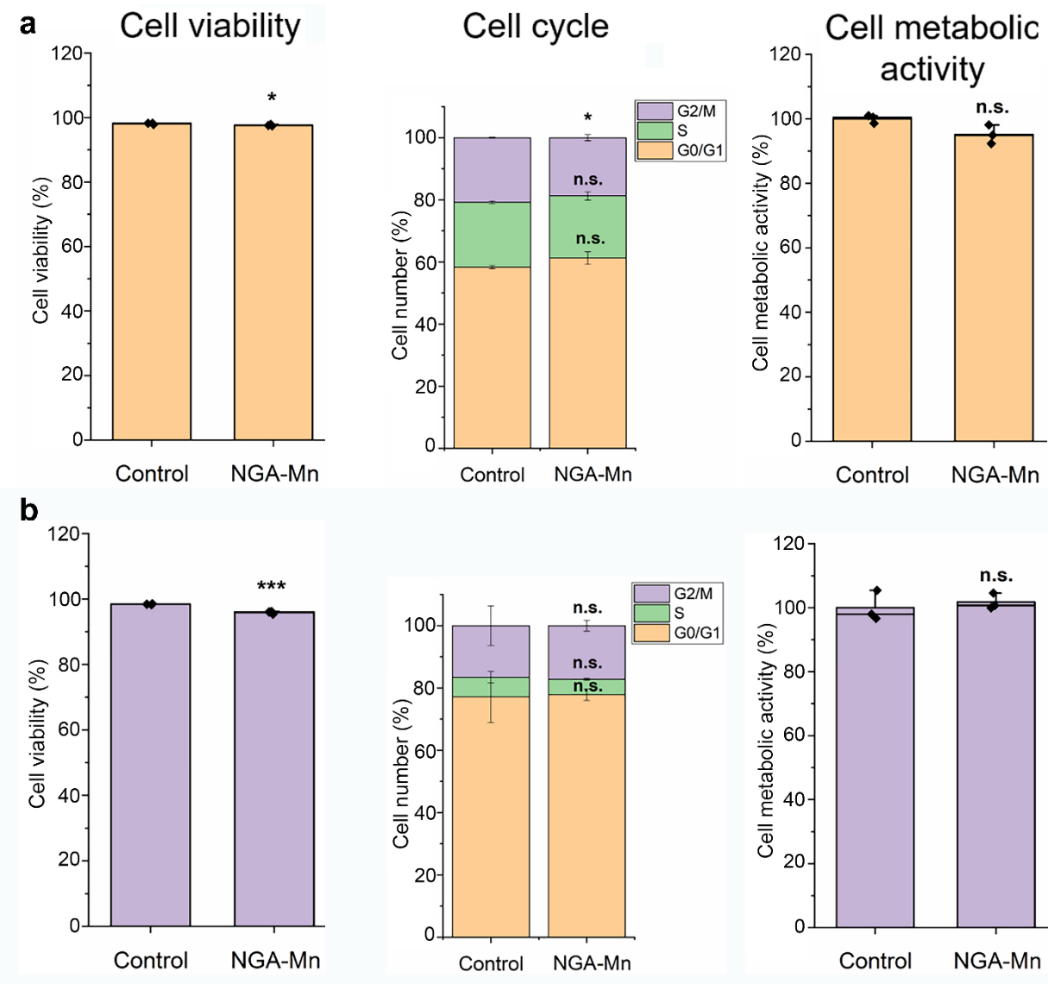


**Figure S13.** Cell viability, cell cycle analysis and cell metabolic activity of (a) THP-1 cells and of (b) BJ skin cells treated with NGA-Mn (cumulative dose of 10 mg L^-1^) on day 15 of long-term experiment. Student-t test was applied for statistical significance (n = 3). ∗p ≤ 0.05, ∗∗∗p ≤ 0.001. Note: n.s. stands for non-significant.


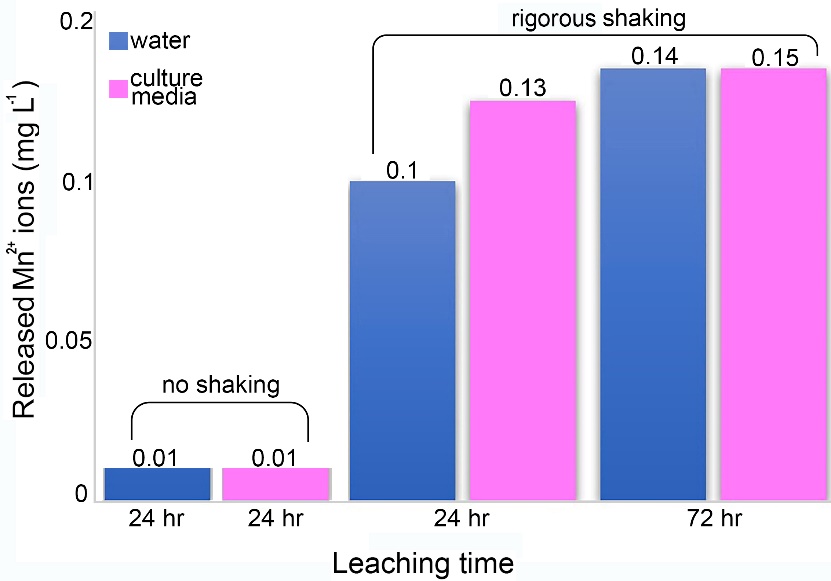


**Figure S14.** Leaching test of manganese from NGA-Mn in water and in cell culture medium after 24, and 72 h. The Mn^2+^ concentrations indicated in the bar graph correspond from left to right to: 0.003, 0.003, 0.03, 0.04, 0.05, and 0.05 % of Mn leached from the total amount of Mn that was initially contained in NGA-Mn which was added in the solution for the leaching test.

**Detailed comments on Figure 4b:** The EPR saturation behaviour has been interpreted by analysing the resonance saturation profile with the following relation, ∫∫*S* = k × √*P* / [1 + (*P*/*P*_1/2_)]^b/2^.^43,44^ ref The factor ∫∫*S* represents the double integrated EPR signal intensity (S), *P* the applied microwave power, *b* the relaxation shape factor (Gaussian line, Lorentzian line or admixture of both), *P*_1/2_ the power at which the signal is half-saturated and *k* is an experimental constant associated with the instrument. The half-saturation power (*P*_1/2_) is a sensitive probe that unveils electronic perturbations of the spin active centers arising from alterations in the system environment, and it is directly connected to the dynamics and perturbation of the quanta being adsorbed and emitted via spin-spin (*T*_2_) and spin-lattice (*T*_1_) relaxation processes (P_1/2_ = α /*T*_1_×*T*_2_, with α = 1/2 *V*/*Qγ*^2^ in which *V* represents the cavity volume, *Q* the cavity quality factor and *γ* the gyromagnetic ratio).

For the unbound (i.e., in the absence of Escherichia coli) form of NGA-Mn, the experimentally determined *P*_1/2_ and b values are *P*_1/2_ = 10.00 mW and *b* = 1.4, and for the bound form of NGA‑Mn interacting with Escherichia coli are *P*_1/2_ = 3.00 mW and *b* = 1.5. From the results, it becomes evident that in the presence of *Escherichia coli* the Mn spin relaxation profile in NGA‑Mn significantly changes (α/*T*_1_× *T*_2_), showing a large decrease in *P*_1/2_ indicating increased disorder (longer *T*_1_) in the Mn ligand-field arising from multiple binding motives. The relaxation shape factor *b* remains nearly unchanged, indicative for the absence of free Mn cations being released from NGA-Mn into the bulk upon interaction with the bacteria. Moreover, we observed that the determined power saturation trends of NGA-Mn in Escherichia coli or in Staphylococcus aureus remained substantially identical to each other. Such similarity of the saturation profiles gives further support to the hypothesis that the interaction of NGA‑Mn with the cell’s surface does not depend on the presence of specific sequences of exposed residues, but rather the system can cooperatively sustain a variety of multiple binding motives (Figure 4g) gated by ligand-field flexibility on the Mn sites.


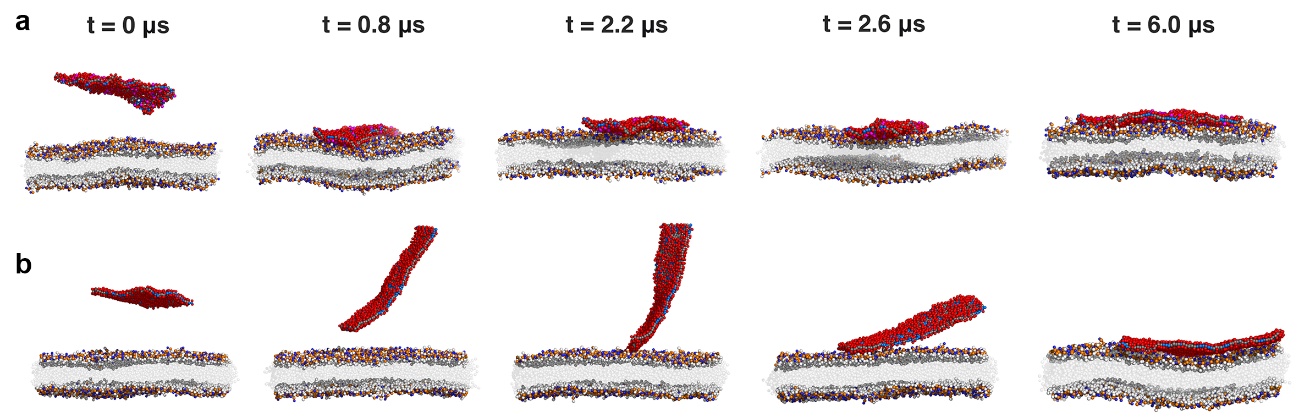


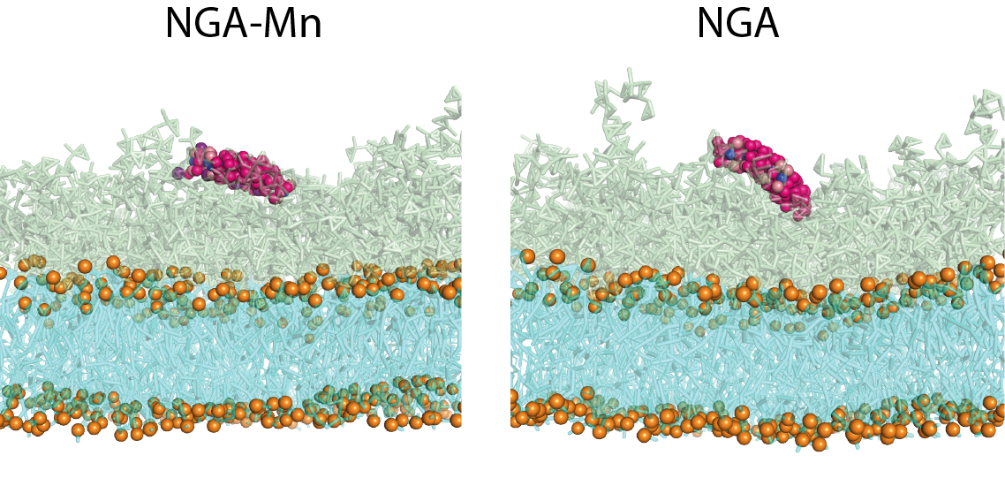


**c**

**Figure S15.** Snapshots from MD simulations of (a) NGA-Mn and (b) NGA. *Further comments on the MD simulations:* The time comparison of both systems shows reduced affinity NGA towards Escherichia coli membrane with the material without Mn^2+^ bivalents approached bacterial more rapidly when compared NGA-Mn. Resulted structures at time 6.0 μs showed similar type of interaction with both materials being completely absorbed upon the bacterial membrane surface. NGA-Mn rapidly approached the membrane surface over the course of first 200 ns of MD simulation. During the next 400 ns of MD simulations, NGA-Mn was adsorbed on the negatively charged surface of Escherichia coli membrane (initial insertion of single NGA and NGA-Mn flakes approximately 4 nm above the membrane). (c) Both NGA-Mn and NGA models rapidly attached to the lipopolysaccharide surface of the bacterial membrane after 1 µs of unbiased simulation. NGA and NGA-Mn models are shown in magenta, lipid bilayer in cyan, polysaccharide layer in green and phosphates are depicted as orange balls. Water and ions are omitted for clarity. Comments: The observation agrees with the hypothesis of NGA-Mn attachment on the polysaccharide surface, but without any noted differences with NGA. Nevertheless, NGA-Mn is a potent antibacterial, but neat NGA not. Since these models (panels a-c) do not account for bonded interactions (i.e., coordination between the metals and lipids or lipopolysaccharides) but only for electrostatics and Lennard-Jones interactions, this is another indication that the antibacterial mechanism is not related with such kind of interactions.


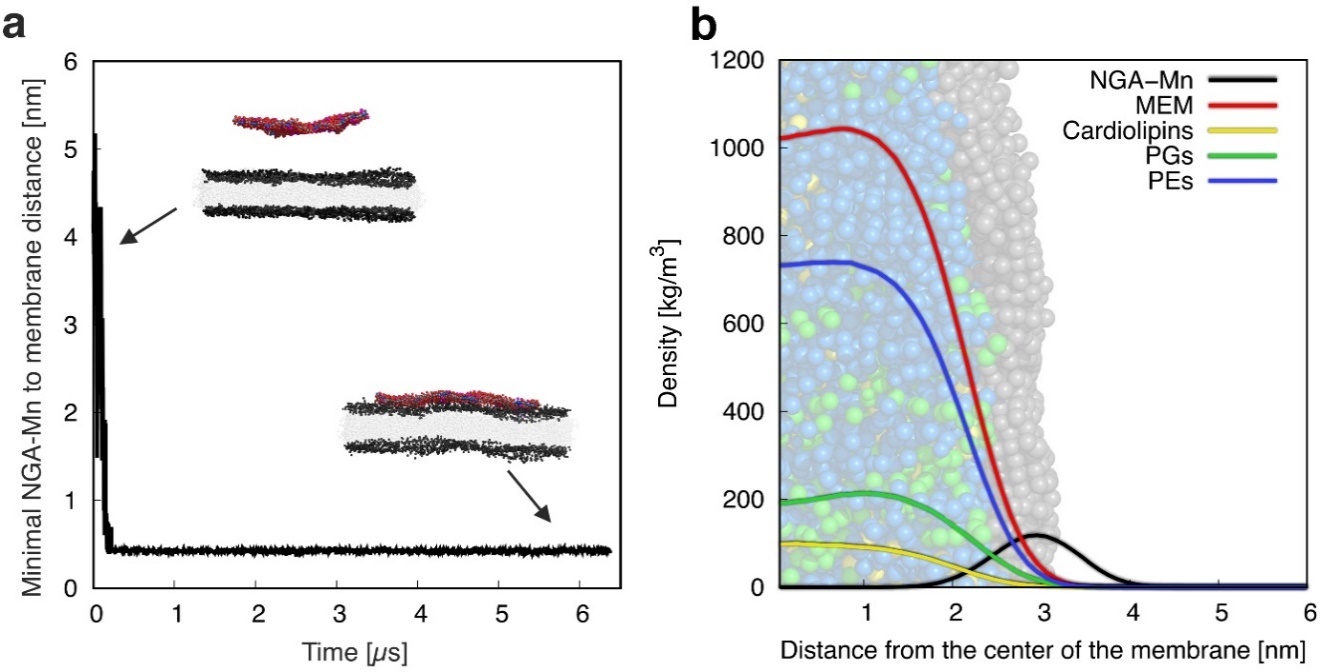


**Figure S16.** (a) Calculated minimal distance of NGA-Mn with respect to the membrane plotted as evolution of time over the course of 3.37 μs showing persistent interaction of the material with *Escherichia coli* membrane. (b) Density profiles centred along the membrane bilayer normal (only leaflet interacting with NGA-Mn was considered) averaged over the last 500 ns of production run. The interaction persisted during the 6.0 μs long simulation with no signs of subsequent detachment, keeping the minimal distance between NGA-Mn and membrane at 0.42 ± 0.02 nm. Individual lines reflect system composition: NGA-Mn – black, overall membrane density – red, cardiolipins – pink, phosphatidylethanolamines – magenta, phosphatidylglycerols – blue.


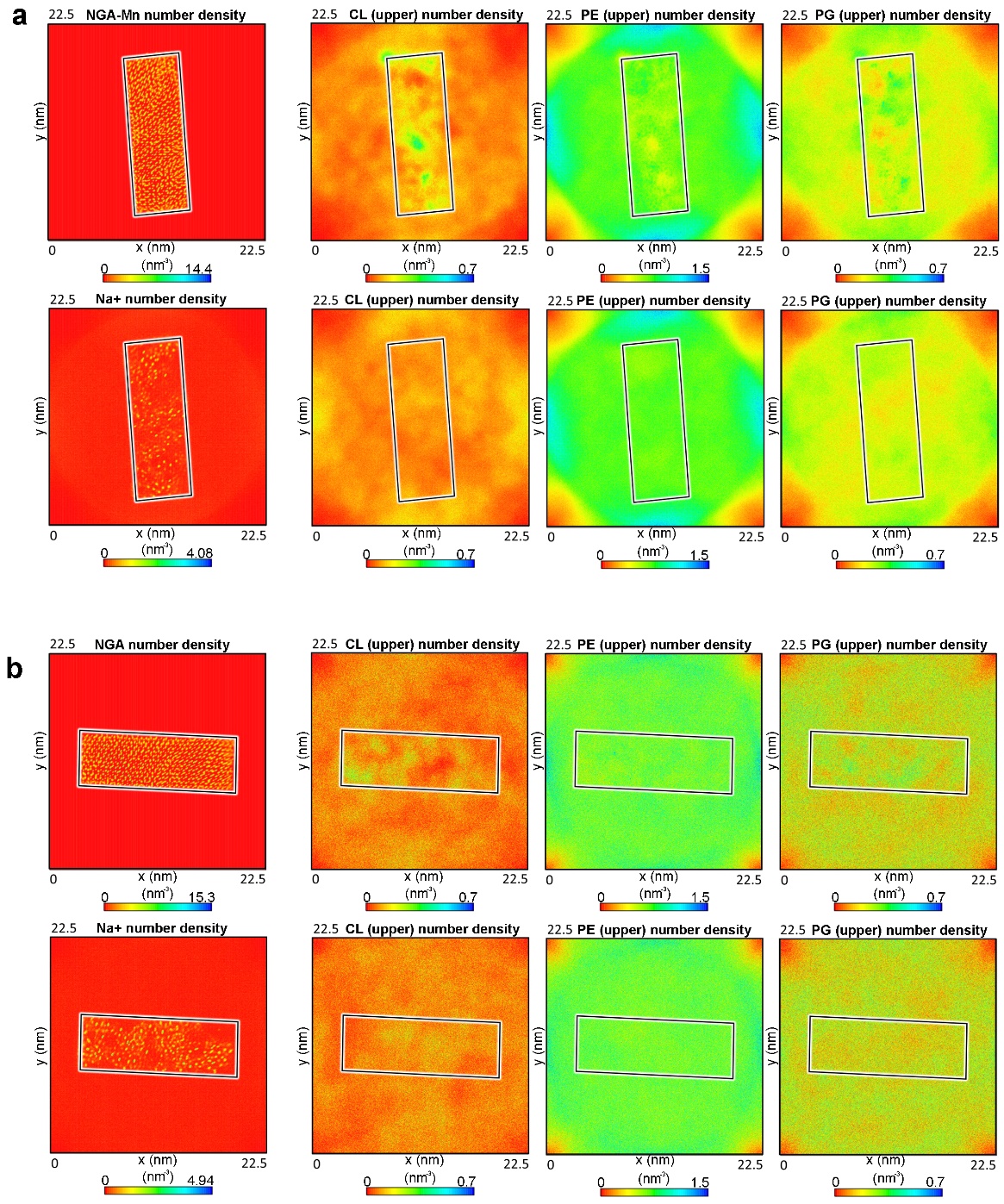


**Figure S17.** 2D density maps for (a) NGA-Mn, and (b) NGA systems fitted at precise position of NGA-Mn and NGA (illustrated as black rectangles). Individual plots show 2D densities of NGA-Mn and NGA (upper left), Na^+^ ions (bottom left) and individual lipid types in upper and lower membrane leaflets: cardiolipins (CL), phosphatidylethanolamines (PEs) and phosphatidylglycerols (PGs). The density plots show the effect of NGA and NGA-Mn on lipids (upper panels) in contrary to random unaffected distributions in lower leaflet (lower panels). Negatively charged lipids (CLs and PGs) are mostly accumulated below NGA-Mn flake. The artificial densities at edges of simulation boxes are consequence of the fitting procedure. NGA-Mn interacted exclusively with polar head-group region of both phospholipids (PEs and PG) and cardiolipins, resulting in accumulation of negatively charged lipids (PGs and CLs) below NGA-Mn flake, whereas NGA without Mn^2+^ showed lower accumulation. Similar results were obtained with other NGA-metal cations, with substantially lower antibacterial activities, suggesting that the activity mechanism of NGA-Mn is not related to electrostatic interactions with the membrane lipids.


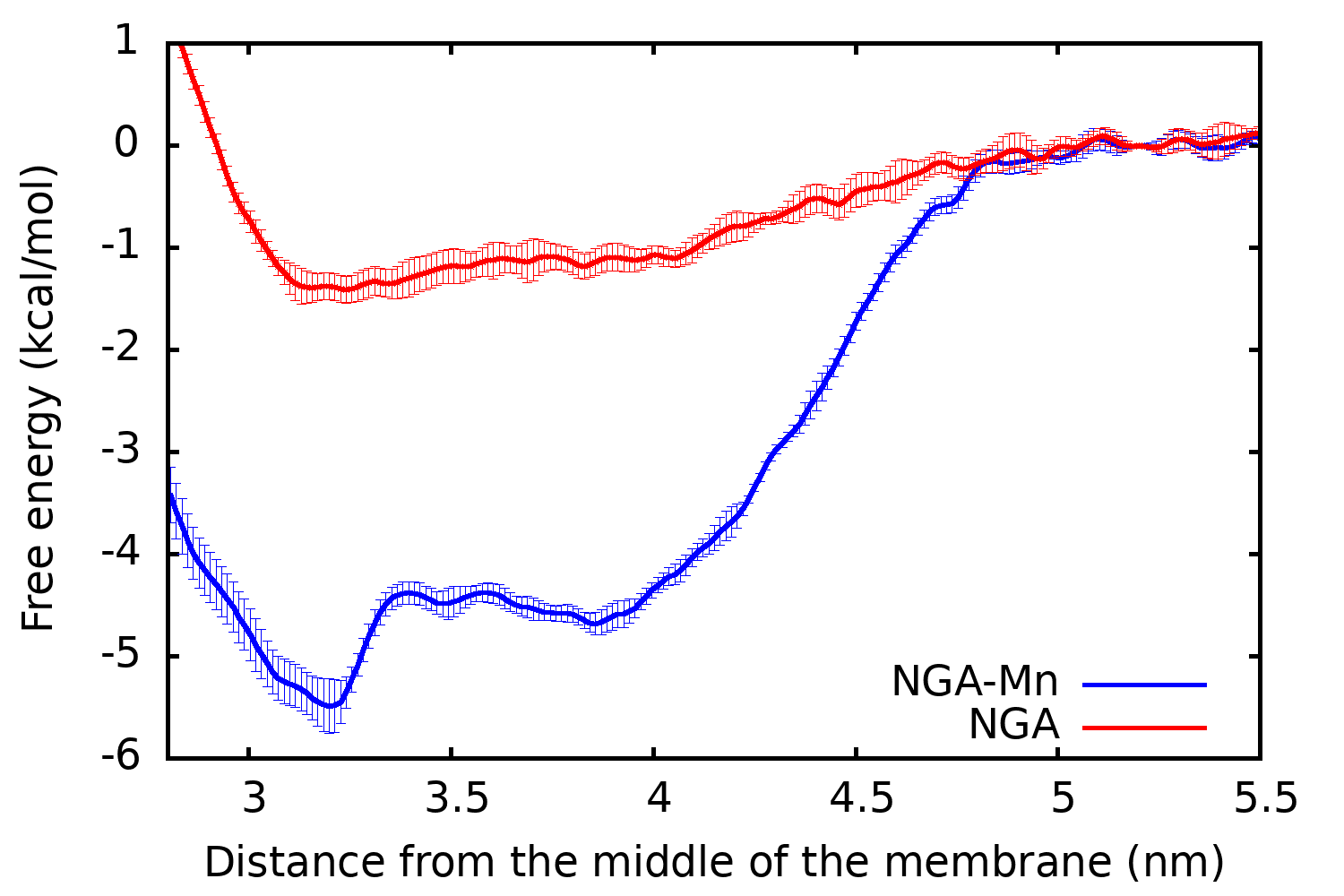


**Figure S18.** Free energy profile of NGA-Mn (blue) and NGA (red) in respect to the distance of NGA and NGA-Mn from the middle of the membrane. The position of the free energy minimum corresponds to the position of graphene flake on the membrane surface, the position at ~5 nm corresponds to graphene in water.

**
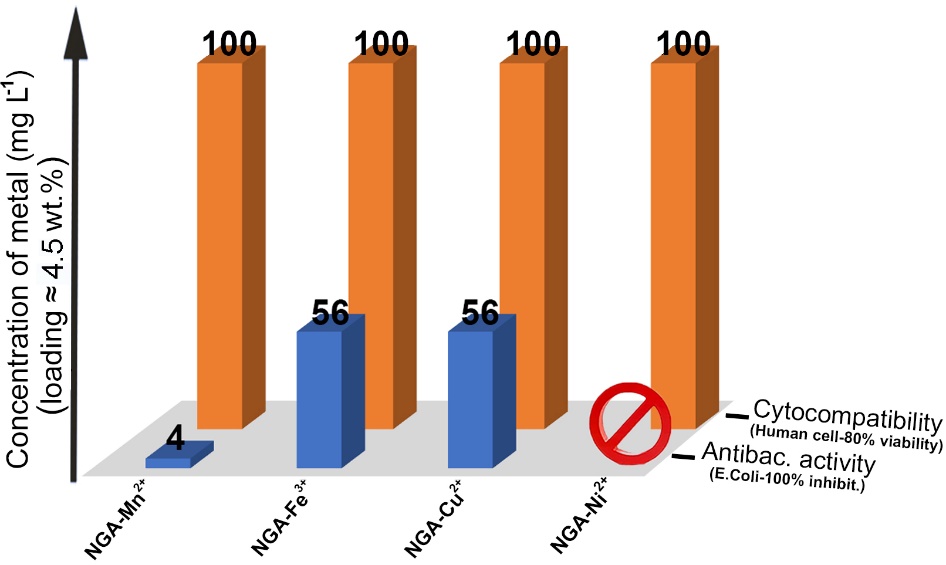
**

**Figure S19.** Comparative graph of antibacterial activity (MIC values) against Escherichia coli and cytocompatibility values (human lung fibroblasts HEL 12469, human skin fibroblasts BJ and human monocyte-like THP-1) for NGA-Mn^2+^, NGA-Fe^3+^, NGA-Cu^2+^, and NGA-Ni^2+^. The metal loading for the NGA‑Mn, NGA‑Fe, NGA‑Cu, and NGA‑Ni products was 4.5, 4.1, 4.4 and 4.7 wt.%, respectively, according to AAS and to ICP-MS. The NGA-Ni material did not show detectable antibacterial activity.


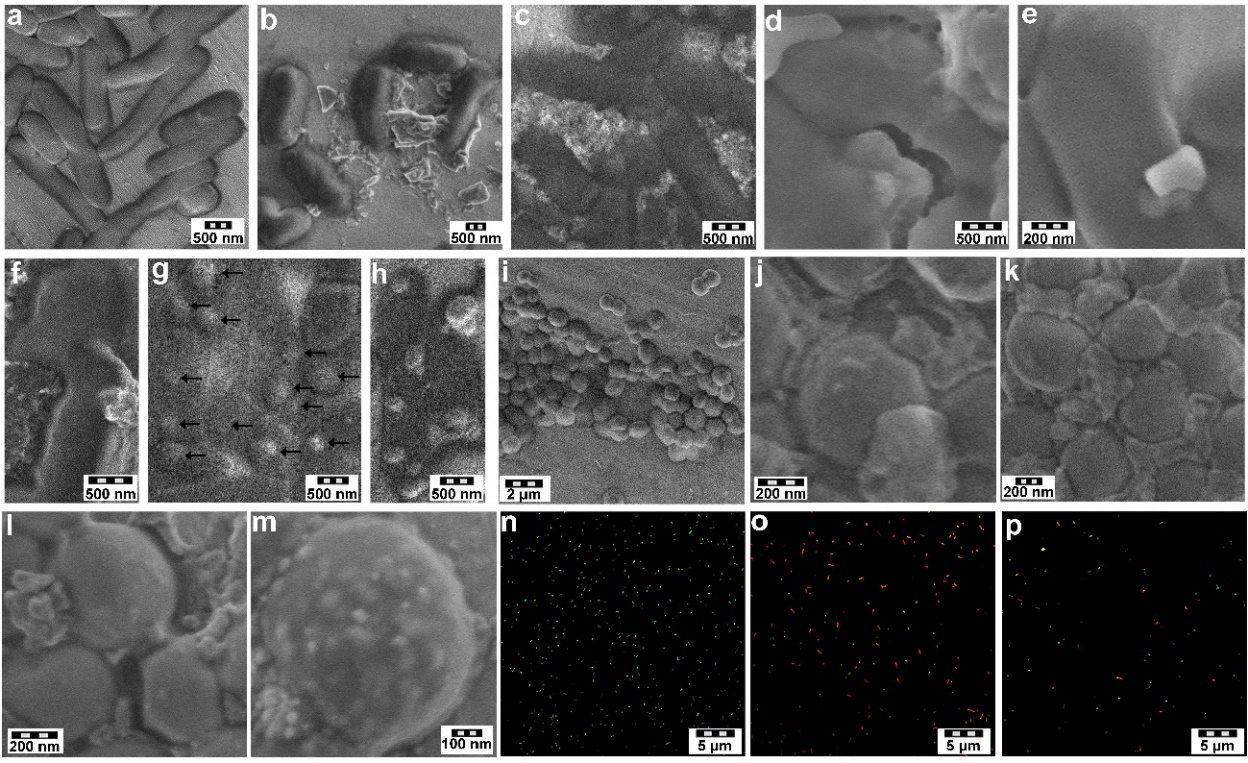


**Figure S20.** SEM image of (a) native *Escherichia coli*, (b, c) treated with bare NGA and (d-h) treated with NGA-Mn at sub-inhibitory concentration. SEM images of methicillin-resistant *Staphylococcus aureus* (MRSA). (i) Native MRSA and (j–m) treated with NGA-Mn. Confocal microscope images of (n) native *Escherichia coli*, (o) control experiment – *Escherichia coli* treated with 70% isopropanol and (p) *Escherichia coli* treated with NGA-Mn. Red staining represents disrupted bacterial membrane. The bacterial cultures treated with NGA-Mn had to be concentrated seven times compared to controls due to the arrest of bacterial division caused in the presence of NGA-Mn.


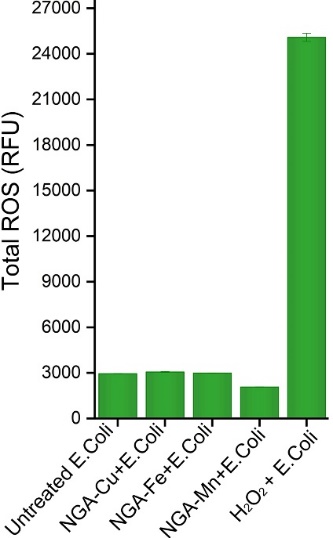


**Figure S21.** Generation of reactive oxygen species (ROS) in *Escherichia coli* without any treatment or treated with NGA hybrid (NGA-Mn, NGA-Cu, NGA-Fe) at a concentration corresponding to MIC_100_. Hydrogen peroxide (2mM, 2h of incubation) was used as a positive control.

**References:**

1. Šedajová, V. *et al.* Nitrogen doped graphene with diamond-like bonds achieves unprecedented energy density at high power in a symmetric sustainable supercapacitor. *Energy Environ. Sci.* **15**, 740–748 (2022).

2. Panáček, D. *et al.* Graphene Nanobeacons with High-Affinity Pockets for Combined, Selective, and Effective Decontamination and Reagentless Detection of Heavy Metals. *Small* **31**, 2201003 (2022).

3. Panáček, A. *et al.* Bacterial resistance to silver nanoparticles and how to overcome it. *Nat. Nanotechnol.* **13**, 65–71 (2018).

4. Pan, Y. *et al.* Antibiotic-Like Activity of Atomic Layer Boron Nitride for Combating Resistant Bacteria. *ACS Nano* **16**, 7674–7688 (2022).

5. Frisch, M. J.; Trucks, G. W.; Schlegel, H. B.; Scuseria, G. E.; Robb, M. A.; Cheeseman, J. R.; Scalmani, G.; Barone, V.; Petersson, G. A.; Nakatsuji, H. *Gaussian16 Revision B.01. Gaussian, Inc.: Wallingford, CT.* (2016).

6. Becke, A. D. Density-functional thermochemistry. III. The role of exact exchange. *J. Chem. Phys.* **98**, 5648–5652 (1993).

7. Grimme, S., Antony, J., Ehrlich, S. & Krieg, H. A consistent and accurate ab initio parametrization of density functional dispersion correction (DFT-D) for the 94 elements H-Pu. *J. Chem. Phys.* **132**, 154104 (2010).

8. Weigend, F. & Ahlrichs, R. Balanced basis sets of split valence, triple zeta valence and quadruple zeta valence quality for H to Rn: Design and assessment of accuracy. *Phys. Chem. Chem. Phys.* **7**, 3297–3305 (2005).

9. Schäfer, A., Huber, C. & Ahlrichs, R. Fully optimized contracted Gaussian basis sets of triple zeta valence quality for atoms Li to Kr. *J. Chem. Phys.* **100**, 5829 (1998).

10. Wadt, W. R. & Hay, P. J. Ab initio effective core potentials for molecular calculations. Potentials for main group elements Na to Bi. *J. Chem. Phys.* **82**, 284 (1998).

11. Marenich, A. V, Cramer, C. J. & Truhlar, D. G. Universal Solvation Model Based on Solute Electron Density and on a Continuum Model of the Solvent Defined by the Bulk Dielectric Constant and Atomic Surface Tensions. *J. Phys. Chem. B* **113**, 6378–6396 (2009).

12. Van Der Spoel, D. *et al.* GROMACS: Fast, flexible, and free. *J. Comput. Chem.* **26**, 1701–1718 (2005).

13. Souza, P. C. T. *et al.* Martini 3: a general purpose force field for coarse-grained molecular dynamics. *Nat. Methods* **18**, 382–388 (2021).

14. Brand, W. *et al.* Interaction of hesperetin glucuronide conjugates with human BCRP, MRP2 and MRP3 as detected in membrane vesicles of overexpressing baculovirus-infected Sf9 cells. *Biopharm. Drug Dispos.* **32**, 530–535 (2011).

15. Parrinello, M. & Rahman, A. Polymorphic transitions in single crystals: A new molecular dynamics method. *J. Appl. Phys.* **52**, 7182–7190 (1981).

16. Jarvis, T. R., Chughtai, B. & Kaplan, S. A. Testosterone and benign prostatic hyperplasia. *Asian J. Androl.* **17**, 212–216 (2015).

17. Paloncýová, M., Langer, M. & Otyepka, M. Structural Dynamics of Carbon Dots in Water and N,N-Dimethylformamide Probed by All-Atom Molecular Dynamics Simulations. *J. Chem. Theory Comput.* **14**, 2076–2083 (2018).

18. Pluhackova, K. & Horner, A. Native-like membrane models of E. coli polar lipid extract shed light on the importance of lipid composition complexity. *BMC Biol.* **19**, 4 (2021).

19. Schrodinger LLC. The PyMOL Molecular Graphics System, Version 1.8. (2015).

20. Gweon, G.-H., Park, J.-G. & Oh, S.-J. Final-state screening effect in the 3 *s* photoemission spectra of Mn and Fe insulating compounds. *Phys. Rev. B* **48**, 7825–7835 (1993).

21. Biesinger, M. C. *et al.* Resolving surface chemical states in XPS analysis of first row transition metals, oxides and hydroxides: Cr, Mn, Fe, Co and Ni. *Appl. Surf. Sci.* **257**, 2717–2730 (2011).

22. Zaoralová, D. *et al.* Tunable Synthesis of Nitrogen Doped Graphene from Fluorographene under Mild Conditions. *ACS Sustain. Chem. Eng.* **8**, 4764–4772 (2020).

23. Wang, X., Bazuin, C. G. & Pellerin, C. Quantitative analysis of hydrogen bonding in electrospun fibers of poly(4-vinyl pyridine)/(4,4′-biphenol) complexes by ATR using liquid blends as models. *Vib. Spectrosc.* **71**, 18–23 (2014).

24. Socrates, G. *G. Socrates, Infrared and Raman Characteristic Group Frequencies: Tables and Charts, 3rd Ed., Wiley, New York, p. 332.* (John Wiley & Sons, 2021).

25. REQUENA, L. & BORNEMANN, S. Barley (Hordeum vulgare) oxalate oxidase is a manganese-containing enzyme. *Biochem. J.* **343**, 185–190 (1999).

26. Meirovitch, E. & Poupko, R. Line shape studies of the electron spin resonance spectra of manganese protein complexes. *J. Phys. Chem.* **82**, 1920–1925 (1978).

27. Whiting, A. K., Boldt, Y. R., Hendrich, M. P., Wackett, L. P. & Que, L. Manganese(II)-Dependent Extradiol-Cleaving Catechol Dioxygenase from Arthrobacter globiformis CM-2. *Biochemistry* **35**, 160–170 (1996).

28. Su, C., Sahlin, M. & Oliw, E. H. Kinetics of Manganese Lipoxygenase with a Catalytic Mononuclear Redox Center *. *J. Biol. Chem.* **275**, 18830–18835 (2000).

29. Tanner, A., Bowater, L., Fairhurst, S. A. & Bornemann, S. Oxalate Decarboxylase Requires Manganese and Dioxygen for Activity: OVEREXPRESSION AND CHARACTERIZATION OF BACILLUS SUBTILIS YvrK AND YoaN *. *J. Biol. Chem.* **276**, 43627–43634 (2001).

30. Li, Y. *et al.* Asymmetric N, O-Coordinated Single Atomic Co Sites for Stable Lithium Metal Anodes. *ENERGY Environ. Mater.* **n/a**, e12449.

31. Li, J. *et al.* Interfacial engineering of Bi2S3/Ti3C2Tx MXene based on work function for rapid photo-excited bacteria-killing. *Nat. Commun.* **12**, 1224 (2021).

32. Zabara, M. *et al.* Multifunctional Nano-Biointerfaces: Cytocompatible Antimicrobial Nanocarriers from Stabilizer-Free Cubosomes. *Adv. Funct. Mater.* **29**, 1904007 (2019).

33. Han, H. *et al.* Inherent Guanidine Nanogels with Durable Antibacterial and Bacterially Antiadhesive Properties. *Adv. Funct. Mater.* **29**, 1806594 (2019).

34. Luo, Y. *et al.* Anti-Infective Application of Graphene-Like Silicon Nanosheets via Membrane Destruction. *Adv. Healthc. Mater.* **9**, 1901375 (2020).

35. Shen, S. *et al.* Synthesis of CaO2 Nanocrystals and Their Spherical Aggregates with Uniform Sizes for Use as a Biodegradable Bacteriostatic Agent. *Small* **15**, 1902118 (2019).

36. Cai, T. *et al.* Optimization of Antibacterial Efficacy of Noble-Metal-Based Core–Shell Nanostructures and Effect of Natural Organic Matter. *ACS Nano* **13**, 12694–12702 (2019).

37. Wang, Z. *et al.* A naturally inspired antibiotic to target multidrug-resistant pathogens. *Nature* **601**, 606–611 (2022).

38. Durand-Reville, T. F. *et al.* Rational design of a new antibiotic class for drug-resistant infections. *Nature* **597**, 698–702 (2021).

39. Smith, P. A. *et al.* Optimized arylomycins are a new class of Gram-negative antibiotics. *Nature* **561**, 189–194 (2018).

40. Shukla, R. *et al.* Teixobactin kills bacteria by a two-pronged attack on the cell envelope. *Nature* **608**, 390–396 (2022).

41. Doern, G. V. & Brecher, S. M. The Clinical Predictive Value (or Lack Thereof) of the Results of In Vitro Antimicrobial Susceptibility Tests. *J. Clin. Microbiol.* **49**, S11–S14 (2011).

42. Belanger, C. R. & Hancock, R. E. W. Testing physiologically relevant conditions in minimal inhibitory concentration assays. *Nat. Protoc.* **16**, 3761–3774 (2021).

43. Zoppellaro, G. *et al.* Modulation of the Ligand-Field Anisotropy in a Series of Ferric Low-Spin Cytochrome c Mutants derived from Pseudomonas aeruginosa Cytochrome c-551 and Nitrosomonas europaea Cytochrome c-552: A Nuclear Magnetic Resonance and Electron Paramagnetic Resonance Study. *J. Am. Chem. Soc.* **130**, 15348–15360 (2008).

44. Tomter, A. B. *et al.* Spectroscopic Studies of the Iron and Manganese Reconstituted Tyrosyl Radical in Bacillus Cereus Ribonucleotide Reductase R2 Protein. *PLOS ONE* **7**, e33436 (2012).
